# Nano‐Flow Cytometry‐Guided Discrimination and Separation of Human Cytomegalovirus Virions and Extracellular Vesicles

**DOI:** 10.1002/jev2.70060

**Published:** 2025-05-02

**Authors:** Vladimir Bokun, Blair L. Strang, Paschalia Pantazi, Yan Liu, Beth Holder

**Affiliations:** ^1^ Institute of Reproductive and Developmental Biology, Department of Metabolism, Digestion and Reproduction, Faculty of Medicine Imperial College London London UK; ^2^ Institute for Infection and Immunity City St George's, University of London London UK

**Keywords:** cytomegalovirus, extracellular vesicles, nano‐flow cytometry, nanoscale flow cytometry, nucleic acid staining, particle purification, virus

## Abstract

Accurate quantification and physical separation of viral particles and extracellular vesicles (EVs) produced by virus‐infected cells presents a significant challenge due to their overlapping physical and biochemical properties. Most analytical methods provide information on a particle mixture as a whole, without distinguishing viral particles from EVs. By utilising nano‐flow cytometry (nFC), a specialised form of flow cytometry adapted for the investigation of nanoparticles, we developed a simple, nucleic acid staining‐based method for discrimination and simultaneous quantification of the human cytomegalovirus (HCMV) virions, dense bodies and EVs, within extracellular particle mixtures produced by HCMV‐infected cells. We show that nucleic acid staining allows for discrimination of the individual particle types based on their distinct fluorescence/side scatter profiles, assessed at single‐particle level by nFC. Following this, we optimised a method for physical separation of EVs from viral particles, based on high‐speed centrifugation through density cushions, using nFC as a tool to evaluate the purity of the isolated EVs. The methods introduced here have the capacity to circumvent common difficulties associated with the co‐investigation of EVs and viruses.

## Graphical abstract

Graphical illustration of the nano‐flow cytometry‐based method for the discrimination of HCMV virions, dense bodies, and extracellular vesicles.

Extracellular particles found in the conditioned medium of HCMV‐infected cells are stained with a nucleic acid dye (such as SYBR Green I), and forced into a single‐particle stream. Single particles are excited at 488 nm and side scattered light and nucleic acid fluorescence measured through 488±5 and 520±20 nm bandpass filters, respectively. Each particle type (HCMV virions dense bodies, extracellular vesicles) is then identified based on its distinct side scatter/fluorescence signature, gated, and quantified using a concentration standard.

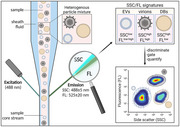

## 1 Introduction

Extracellular vesicles (EVs) are lipid‐delimited particles released by cells as a mode of inter‐cellular communication (Alenquer and Amorim 2015). EVs have been a subject of growing interest in many research areas, including viral infection (Alenquer and Amorim 2015; Sadeghipour and Mathias 2017; Anderson et al. 2016; Hoen et al. 2016; Raab‐Traub and Dittmer 2017). EVs play important roles in infection by viruses of multiple classes, acting in a pro‐ or anti‐viral fashion, depending on the virus, cell type, stage of infection, and other factors (Alenquer and Amorim 2015; Sadeghipour and Mathias 2017; Anderson et al. 2016; Hoen et al. 2016; Raab‐Traub and Dittmer 2017). A prominent example of this is the link between EVs and the herpesvirus human cytomegalovirus (HCMV), wherein HCMV co‐opts cellular machinery involved in EV biogenesis to produce viral particles and enhance HCMV replication (Streck et al. 2020; Turner et al. 2020; Streck et al. 2018). Various other viruses have also been described to utilise the cellular EV biogenesis pathways, leading to incorporation of host cell components into mature viral particles, as reviewed elsewhere (Maxwell and Frappier 2007). Conversely, the cargo of EVs can be altered in response to viral infection and can include viral glycoproteins enriched at the sites of EV biogenesis (Sadeghipour and Mathias 2017; Anderson et al. 2016; Hoen et al. 2016; Raab‐Traub and Dittmer 2017). Potential implications of such phenomena are numerous and at present poorly understood.

EVs are present in all unpurified in vitro viral preparations, so the study of their functional properties is impossible without their separation from viral particles and contaminants. This can be challenging due to the similarities in biochemical and physical features (size and density) of EVs and viral particles (Zhou and McNamara 2020). Techniques exploiting differences in these properties for purification may not yield sufficiently pure EVs or virions (Metzner and Zaruba 2021; McNamara and Dittmer 2020). For instance, similar density or size of viruses and EVs, or their aggregation and co‐migration, could prevent a complete separation of particles by density gradient ultracentrifugation, and contamination is often not checked rigorously (Zhou and McNamara 2020; McNamara and Dittmer 2020). Additionally, ultracentrifugation can cause physical alterations or damage to the particles under study (McNamara and Dittmer 2020; Mol et al. 2017; Trépanier et al. 1981; Soo‐hyun and Kwang‐il 2017; Stinski 1976). Other methods, such as magnetic capture or affinity chromatography, are not always selective, due to the overlap between protein compositions of EVs and viruses.

The challenges associated with the purification of viruses and EVs can be compounded by the shortcomings in some of the routinely employed methodology for their characterisation, which can lead to misinterpretations of the levels of contamination in purified EV or virus preparations. Methods such as ELISA, western blotting and proteomics allow for global readouts reflective of a particle mixture as a whole, while single‐particle characterisation methods, like electron microscopy, can be unavailable and have low throughput. Finally, the viral plaque assay used for assessing HCMV infectivity is based on the presence of functionally intact virus, and therefore cannot detect contamination with viral particles rendered non‐infectious during purification.

Recently, a specialised form of flow cytometry designed for nano‐scale use, herein referred to as nano‐flow cytometry (nFC), has been applied for the detection, quantification and phenotyping of EVs, as well as other nanoparticles, with improved detection sensitivity and sizing accuracy (Dong et al. 2020; Ma et al. 2016; Niu et al. 2021; Tian et al. 2020). The method incorporates adaptations that prevent coincident particle detection (Lucchetti et al. 2020), and possesses highly sensitive optical detection that is suited to low light scatter and fluorescence signals associated with EVs and viruses.

Here, we designed nFC‐based methods to achieve full discrimination and simultaneous quantification of HCMV particles and associated EVs, in complex extracellular particle mixtures produced by HCMV‐infected cells. We also report an optimised protocol for the purification of EVs from HCMV‐infected cells, based on differential viral particle/EV densities, guided by nFC as a purity screening tool. Collectively, these methods represent a framework for analysing and purifying HCMV‐infected cell EVs and viral particles for functional or biochemical studies, with potential applicability to other viruses.

## 2 Materials and Methods

### 2.1 Viruses and Cells

Low passage (< 6) human cytomegalovirus (HCMV) strain Merlin R1111 (with deletions in open reading frames encoding RL13 and UL131 to allow cellular release of virus) was used in all experiments (Stanton et al. 2010). Virus stocks were generated from human foreskin fibroblasts (HFFs) (clone Hs27; ATCC) cultured in Dulbecco's modified Eagle medium (DMEM, Gibco) supplemented with 10% fetal bovine serum (FBS), 100 U/mL penicillin and 100 µg/mL streptomycin (all from Gibco). Virus stocks were produced by infecting confluent HFF monolayers at the multiplicity of infection (MOI) of 0.01, and collecting supernatants when all cells displayed cytopathic effects and were detaching from the flasks. Supernatants were clarified by low‐speed centrifugation (400 × *g* for 10 min) and stored at −80°C.

### 2.2 Infection of HFF Cells and Generation of Conditioned Medium

Confluent HFFs were left uninfected or were infected at 0.01 MOI, by allowing the virus to enter the cells for 1 h at 37°C. The virus stock was then replaced with complete DMEM containing 10% (except where indicated) commercial exosome‐depleted fetal bovine serum (from Gibco; catalogue number A2720801) and antibiotics penicillin and streptomycin (as above). Conditioned medium was then collected when all cells displayed cytopathic effects, clarified by centrifugation at 300 × *g* for 5 min and 3000 x g for 20 min (except where indicated), and then either analysed in that form or subjected to additional processing.

### 2.3 Plaque Assay to Determine Viral Titer

HFFs were seeded in 24‐well plates at 50,000 cells per well and infected with 10‐fold serially diluted viral stocks or experimental samples one day after seeding (in triplicate). After 1 h of adsorption, viral suspensions were aspirated from the plates and cells overlayed with 1.2% methylcellulose (Sigma‐Aldrich) in DMEM supplemented with 2% FBS and penicillin and streptomycin (all from Gibco). After 14–21 days of incubation, overlay medium was aspirated and cells fixed with methanol for 30 min at room temperature. Crystal violet (1%) was added to fixed cell monolayers for 30 min, and then washed three times with distilled water to visualise the plaques. Plaque‐forming units per millilitre (PFU/mL) were calculated by multiplying average plaque counts by the dilution factor and dividing by the volume of viral suspension used to infect the cells.

### 2.4 Nano‐flow Cytometry

#### 2.4.1 Instrument Setup and Calibration

Nano‐flow cytometry was performed using the flow nanoanalyser instrument manufactured by nanoFCM, operated by NF Profession software (versions 1.11 or 2.12). The instrument was adjusted before each use using quality control silica nanospheres with a diameter of 250 nm (from nanoFCM), diluted 100‐fold in ultrapure water. Quality control nanospheres were used to calibrate the optics, including the filter and laser positioning, and served as the concentration standard by which experimental particles were quantified. Excitation was provided by 488‐ and 638‐nm lasers (at 10 kW/50 kW and 10 kW/100 kW, respectively, for all experimental samples) and data collected through a 488 ± 5 nm bandpass filter for side scatter and 525 ± 20 nm or 670 ± 15 nm bandpass filters for fluorescence. All experimental and quality control samples were acquired at the sampling pressure of 1.0 kPa for 1 min. Data processing involved in generating particle size distributions and concentrations was done in the accompanying software.

#### 2.4.2 Thresholding

The thresholding was performed using the automatic threshold capability of the included software, which was set to ‘small signal’. This takes into account the mean and the standard deviation of the negative event signal intensity, and applies the following formula to set the threshold: mean + SD × 3, (SD = standard deviation). Manual thresholding was performed for CD81 antibody staining of purified EVs.

#### 2.4.3 Sample Processing for nFC

All samples were first fixed with 4% paraformaldehyde (Pierce) for 20 min at room temperature (except where indicated), and then diluted at least 10‐fold in Dulbecco's phosphate‐buffered saline (DPBS; Lonza), to yield 2000–12,000 events per 1‐min acquisition. DPBS was used as the blank.

#### 2.4.4 Sizing

Sizing was performed based on side scatter intensities of silica nanosphere sizing standard sets (S16M‐Exo—68 nm, 91 nm, 113 nm, 155 nm; and S17M‐MV—155 nm, 298 nm, 535 nm, 850 nm; both obtained from nanoFCM) excited at 488 nm and measured through the 488 ± 5 nm filter. Standard curves were constructed from side scatter intensities and experimental particle diameter determined in the included software. S16M‐Exo sizing standards were used in all experiments, while the larger S17M‐MV sizing standards were used for sizing performance comparisons only. In these comparisons, side scatter decay settings of 10%, 2% and 0.2% were also compared. Additionally, extracellular particles from uninfected and HCMV‐infected cells prepared by SEC were treated with either 1% Triton X‐100 (Sigma‐Aldrich) or DPBS (as control) for 1 h on ice, followed by dilution in DPBS containing 1X SYBR Green I, and size distributions of complete mixtures or gated virions with and without Triton X‐100 treatment generated.

#### 2.4.5 Nucleic Acid Staining‐Based Discrimination of HCMV/DBs/EVs

Conditioned media from uninfected and HCMV‐infected cells was first stained with SYTO 13 at a range of concentrations (100 nM–10 µM), to identify the optimal concentration. Median fluorescence intensities (MFIs) were recorded, and the stain index calculated for each SYTO 13 concentration (MFI^pos^–MFI^neg^) / (SD^neg^ × 2). In subsequent experiments, extracellular particle nucleic acids were stained using 2 µM SYTO 13 (Invitrogen), 1X SYBR Green I (MedChemExpress) or 1X SYBR Safe (Invitrogen), following 4% paraformaldehyde fixation (for 20 min), and analysed by nFC. Stained particles were excited by the 488‐nm laser (at 10 mW), and emitted fluorescence measured through the 525 ± 20 nm filter. HCMV virion quantification was performed by gating on the virion population in the NF Profession software. The MFIs of the gated virion populations were obtained in the included software.

### 2.5 Size Exclusion Chromatography (SEC)

Clarified conditioned medium from uninfected and infected HFFs was filtered through 0.45‐µm polyethersulfone (PES) syringe filters (Millipore) and concentrated using 30‐kDa ultrafiltration columns (Vivaspin, from Sartorius). 0.5 mL of concentrated medium was applied to qEV original columns with a pore size of 70 nm (IZON), and the first three 0.5‐mL fractions following void volume (2.5 mL) collected in DPBS, using an automated fraction collector (IZON). These fractions were pooled and stored at ‐80°C.

Alternatively, to process larger volumes, custom SEC columns were made by packing BioRad glass Econo‐Columns (2.5‐cm diameter) with Sepharose CL‐2B (Cytiva) to approximately 22 cm, constituting a bed volume of about 108 mL. A total of 2.5 mL of ultrafiltration‐concentrated EVs were applied to the column and 40 3‐mL fractions collected. Samples were eluted in DPBS and fractions containing the highest EV concentrations (fractions 10‐15) were pooled and concentrated by ultrafiltration (30 kDa) before storage at −80°C.

### 2.6 Transmission Electron Microscopy (TEM)

A total of 4 µL of particle suspensions purified by SEC were deposited onto 300 mesh carbon support grids (Agar Scientific) previously glow‐discharged by plasma for 50 s using a Fischione NanoClean Model 1070. After 5 min of adsorption, excess buffer was blotted off and grids briefly washed and incubated with 2% uranyl acetate for 45 s. Excess liquid was blotted off and grids left to dry at room temperature prior to imaging. Samples were screened on an FEI Tecnai T12 Spirit with the accelerating voltage of 120 kV, at 6500× and 21,000× magnifications. Sizing of viral particles was performed using ImageJ.

For cryo‐EM visualisation of HCMV virions, 4 µL of gradient‐purified viral particles (as described below) were deposited onto glow‐discharged quantifoil holey carbon grids, and plunge‐frozen in liquid ethane cooled by liquid nitrogen, using a Vitrobot (ThermoFisher). Plunge‐frozen grids were imaged on an FEI T20 Spirit with 200 kV accelerating voltage. Sizing of viral particles was performed using ImageJ.

### 2.7 Purification of HCMV Virions by Gradient Ultracentrifugation

Extracellular particles from HCMV‐infected HFF clarified conditioned medium were centrifuged at 25,000 × *g* for 2 h at 4°C in a JS‐13.1 swing bucket rotor (Beckman). The resulting pellet was resuspended in 0.5 mL of residual buffer and loaded onto a discontinuous gradient comprising 18%, 20%, 22%, 24% and 26% iodixanol (OptiPrep; Sigma‐Aldrich) in DPBS with 10 mM EDTA. The gradient was ultracentrifuged for 150 min at 44,000 rpm at 4°C in an MLS‐50 swing bucket rotor (Beckman) without braking. Particles located on top of the 22% layer were collected and centrifuged at 32,000 rpm for 70 min, in the MLS‐50 rotor, at 4°C, and resuspended in residual DPBS. The sample was kept at −80°C until it was analysed by cryo‐EM. The gradient‐purified particles were fixed with 4% paraformaldehyde for 20 min before cryo‐EM.

### 2.8 Quantitative Polymerase Chain Reaction (qPCR)

Clarified conditioned medium (stored at –80°C) was first subjected to digestion with DNase I (NEB) per manufacturer's instructions. Viral DNA extraction was then performed using the PureLink Viral RNA/DNA mini kit (Invitrogen). WHO international HCMV reference standard, containing a known quantity of HCMV international units (IUs), was obtained from the National Institute of Biological Standards and Control (NIBSC) (Fryer et al. 2016), and subjected to the same processing as the experimental samples, to generate a standard curve. Samples and standards were analysed using a commercially available TaqMan probe and primer set (Applied Biosystems assay ID Vi06439643_s1) and iTaq Universal Probes Supermix (Biorad), on a StepOnePlus real time qPCR machine (Applied Biosystems). The viral genome copy numbers in the conditioned medium were then back‐calculated based on the obtained values and the volume of the starting input material.

### 2.9 Separation of EVs From Viral Particles by High‐Speed Centrifugation Through Iodixanol Cushions

Conditioned medium was clarified by centrifugation at 300 × *g* for 5 min and 3000 × *g* for 20 min, followed by filtration through 0.45‐µm PES filters. Clarified conditioned medium was then transferred into high‐speed centrifuge bottles (35 mL per bottle) (Oak Ridge) and 16% iodixanol (OptiPrep) in DPBS was then underlaid (5 mL per bottle) as a cushion underneath the particle suspension. Bottles were centrifuged at 25,000 × *g* in a Beckman JS‐13.1 swing bucket rotor (with a k‐factor of 1841) for 150 min at 4°C with the slowest acceleration and no braking. The resulting supernatants, including the interface, were collected for EV isolation by SEC, using a self‐packed Sepharose column described previously, while the pellets were saved separately. In addition to the HFF cell conditioned medium, non‐conditioned complete medium (DMEM with 10% exosome‐depleted FBS) was subjected to the same processing to confirm the absence of contaminating particles.

### 2.10 Nanoparticle Tracking Analysis (NTA)

To determine EV concentrations in SEC fractions, fractions 1–30 were diluted in DPBS and 1 mL injected into a ZetaView PMX 120 S NTA instrument (ParticleMetrix) equipped with a 520‐nm laser and run by the accompanying ZetaView 8.05.12 SP2 software. The instrument was adjusted before each use using 100‐nm nanospheres (Item 3100A, ThermoFisher) diluted 1:250,000 in ultrapure water. Samples were analysed by capturing a 21‐s video, which included 11 positions, with two readings of each position, followed by an automated concentration calculation in the included software. The instrument pre‐acquisition parameters were: temperature of 23°C, sensitivity of 75, frame rate of 30 frames per second, shutter speed of 100, and laser pulse duration equal to that of shutter duration. Post‐acquisition parameters were: minimum brightness of 30, maximum size of 1000 pixels, a minimum size of 10 pixels and a trace length of 15.

### 2.11 Protein Assay

SEC fractions were analysed for protein concentration using the Bradford assay (BioRad), per manufacturer's instructions for the microplate microassay. This was done by mixing 150 µL of the included reagent with 150 µL of each diluted or undiluted sample for 5 min, and reading absorbance at 595 nm using a Spectramax microplate reader (Molecular Devices). A 20–0.625 µg/mL standard curve of bovine serum albumin (Pierce) was prepared in DPBS, and standard curves generated in the included software, Softmax Pro (Molecular Devices). DPBS was used as the blank. All samples and standards were assayed in triplicate.

### 2.12 Silver Staining

A total of 15 µL of SEC fractions 6–35 were mixed with 10 × 3‐((3‐cholamidopropyl) dimethylammonio)‐1‐propanesulfonate (CHAPS) (Sigma‐Aldrich) buffer (1% CHAPS, 25 mM Tris, 150 mM sodium chloride (NaCl), 5 mM EDTA in ultrapure water, pH = 7.4) and 6X lithium dodecyl sulfate (LDS) reducing sample buffer (Alfa Aesar) and heated at 95°C for 5 min. Protein samples were then centrifuged briefly and loaded onto 1.0‐mm Bolt Bis‐Tris Plus 4%–12% gradient SDS‐PAGE mini 17‐well gels (Invitrogen), and run at 200 volts for 32 min, in MOPS‐SDS running buffer (Invitrogen). SDS‐PAGE gels were then stained using the Pierce Silver staining kit, per manufacturer's instructions, and digitised using the ImageQuant LAS 4000 imager (GE Healthcare).

### 2.13 EV Solid Phase Binding Assay

SEC fractions were diluted 3‐fold with DPBS, and dispensed (in duplicate) into a half‐area high‐binding 96‐well plate (Greiner Bio‐One), overnight. All steps were performed at room temperature except where indicated. Adsorbed EVs were fixed with 4% paraformaldehyde for 5 min, and washed three times with DPBS. EVs were then permeabilised (for beta‐actin detection) for 10 min with 0.1% Triton X‐100 in DPBS, or left in DPBS (for CD63). Permeabilised EVs were then washed three times with DPBS. Blocking was performed with 1% bovine serum albumin (BSA; from Sigma‐Aldrich) in DPBS for 1 h. Primary antibodies raised against CD63 (clone MEM‐259; BioRad item MCA2142) or beta‐actin (clone AC‐74; Sigma‐Aldrich item A2228‐100UL) were diluted in blocking buffer to 1 and 2 µg/mL, respectively, and added overnight at 4°C. Three DPBS washes were performed and an anti‐mouse‐Horseradish Peroxidase (HRP) secondary antibody (Dako/Agilent item P044701‐2) diluted to 0.4 µg/mL in blocking buffer added for 2 h. Three DPBS washes were performed and 3,3', 5,5;‐tetramethylbenzidine (TMB) chromogen solution added for 15 min (Abcam). Colour development was stopped by the addition of 0.5 M hydrochloric acid. Absorbance was read at 450 and 650 nm (the latter for background subtraction) using a Tecan Sunrise microplate reader.

### 2.14 CD81 labelling

EVs isolated from uninfected and HCMV‐infected HFF cells were incubated with a recombinant anti‐CD81‐allophycocyanin (APC) antibody (Miltenyi Biotec item 130‐119‐787; RRID:AB_2751844), or isotype control‐APC (Miltenyi Biotec item 130‐113‐434; RRID:AB_2733447), at 1/100 final dilution in 20 µL of DPBS, for 1 h at room temperature. EVs were then adjusted to 100 µL with DPBS and subjected to a clean‐up step using Exo‐spin mini columns (Cell Guidance Systems). Finally, EVs were analysed on the nanoFCM flow nanoanalyser by excitation at 638 nm and fluorescence measured through the 670 ± 15 nm bandpass filter. Thresholds were set identically for uninfected and infected HFF EV pairs.

### 2.15 Statistical Analysis

All statistical testing and chart generation was performed using GraphPad Prism 9. Ratio paired t‐test was performed to compare the extracellular particle concentration in uninfected and HCMV‐infected HFF conditioned media. Differences were considered significant when *p* values were < 0.05.

### 2.16 Reporting Frameworks

Relevant information pertaining to the nFC‐based protocol for the discrimination of HCMV virions and other particles was included in the MIFlowCyt‐EV (Welsh et al. 2020) and the accompanying MIFlowCyt forms (Lee et al. 2008).

EVs purified from HCMV‐infected cells and uninfected cells were characterised in line with the Minimal information for studies of EVs 2018 (MISEV2018) guidelines (Théry et al. 2018). All relevant information pertaining to the isolation protocol as well as the subsequent characterisation of EVs was submitted to the EV‐TRACK knowledgebase (Van Deun et al. 2017), with a resulting score of 71% (96th percentile of all experiments on the same sample type at the time of submission). The EV‐TRACK ID associated with this study is EV230065.

## 3 Results

### 3.1 Side Scatter‐Based Determination of HFF Extracellular Particle Concentration and Size by nFC

**FIGURE 1:**
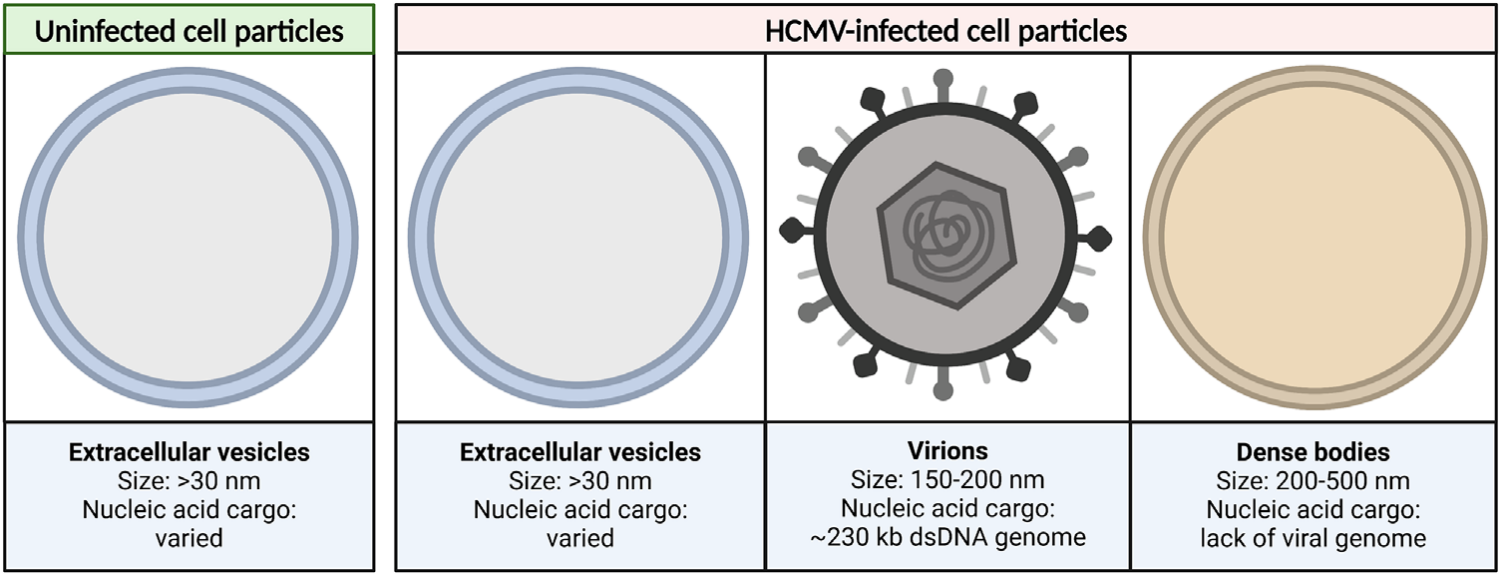
Graphical representation of the main extracellular particle types produced by uninfected and HCMV‐infected HFF cells. Created in BioRender. Bokun, V. (2025) https://BioRender.com/c88l755.

nFC was first used to perform label‐free concentration and size measurements of particles secreted into HFF cell culture media, without their purification. The main types of particles that are expected to be found in conditioned medium of uninfected and HCMV‐infected cells are shown in Figure 1. Particle size was estimated based on side scatter intensities, after applying sizing calibration, while particle concentration was determined using a concentration standard. All standards as well as blank samples are outlined in Figure S1.

**FIGURE 2:**
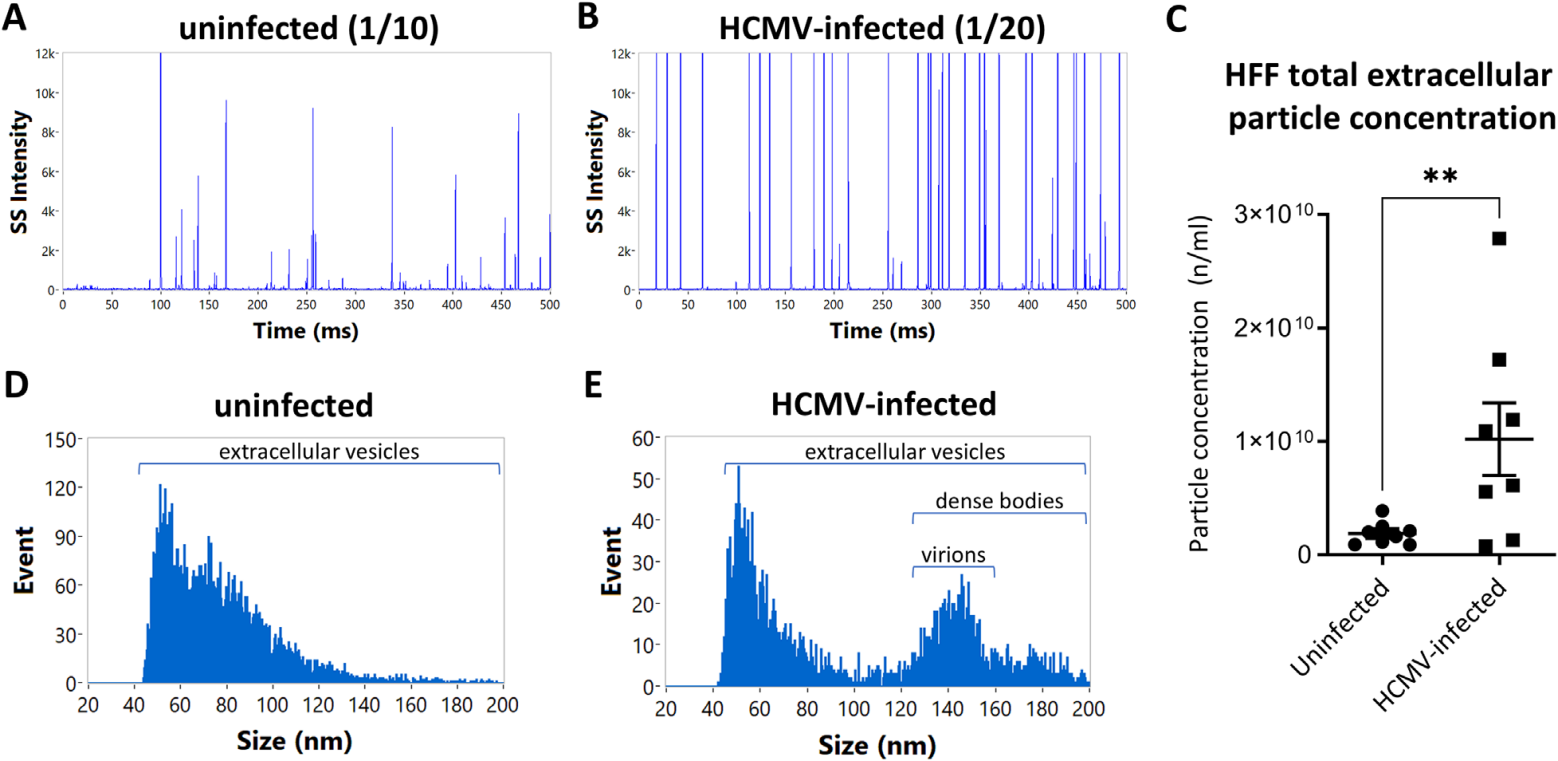
Label‐free particle concentration and size measurements in uninfected and HCMV‐infected HFF conditioned media. Conditioned medium was collected from uninfected and HCMV‐infected HFF cells, clarified by centrifugation, fixed with 4% PFA and diluted in DPBS before being analysed by nFC. Particles were excited at 488 nm and side scatter signal measured through a 488 ± 5 nm filter. Particle concentration was determined based on a concentration standard comprising silica nanospheres of a defined concentration. Particle size was estimated based on side scatter intensities, using silica nanosphere sizing standards. Calibration materials and control samples are shown in Figure S1. (A and B) Representative burst trace plots of the uninfected (A) and HCMV‐infected (B) HFF conditioned medium particles (sample dilutions are indicated in parentheses). Pulses of high side scatter were more frequently seen in HCMV‐infected HFF conditioned media. (C) A scatter plot of the particle concentrations in uninfected and HCMV‐infected HFF conditioned media (mean ± SEMs; two‐tailed ratio *t*‐test, *p* = 0.001). (D and E) Representative size distributions of particles in the uninfected HFF (D) and HCMV‐infected (E) HFF conditioned medium. EV peaks were observed in both samples at about 50 nm, while HCMV virions were observed as a peak at about 140 nm, only from infected cells. Data are from eight experiments. SS = side scatter; ms = milliseconds; nm = nanometers.

Single particles were visualised as pulses of side scatter in burst trace plots (Figure 2A,B), with the intensities of side scatter proportional to the particle size, and the frequency of pulses indicative of the particle concentration. We observed more particles in the burst trace plots of HCMV‐infected HFF conditioned media (Figure 2B) than in the uninfected conditioned media (Figure 2A), and the former were also characterised by more pulses with high side scatter intensities (Figure 2B), as expected based on the presence of the large viral particles produced by infected cells. Using the calibration materials (Figure S1), the side scatter signals were converted to particle concentrations (Figure 2C) and size distributions (Figure 2D,E). Particle concentrations were higher in infected cell conditioned media (Figure 2C). Size distributions of both uninfected (Figure 2D) and HCMV‐infected (Figure 2E) HFF extracellular particles displayed a peak at around 50 nm, which tapered off with increasing diameter. An additional peak was detected at around 140 nm, only in infected HFF conditioned media. This peak was consistent with HCMV virions and dense bodies (DBs) (Figure 2E). Dense bodies are HCMV infection‐induced genome‐deficient subviral particles characterised by a similar but wider size range than HCMV virions (Pepperl et al. 2000; Talbot and Almeida 1977).

The serum used for medium supplementation can contain lipoprotein and protein aggregates, which could be detected by nFC (Urzì et al. 2022). Therefore, to ensure that the detected particles did not originate from the medium itself, we used exosome‐depleted FBS, and subjected the non‐conditioned, complete DMEM medium to the same nFC analysis. The concentration of particles in the non‐conditioned medium was on average 41‐ and 220‐fold lower than in the uninfected and HCMV‐infected HFF conditioned medium, respectively, confirming that the medium itself did not contribute appreciably to the measured experimental particle concentrations (Figure S1D).

Taken together, these experiments showed that nFC could detect and quantify particles in the clarified condition medium from HCMV‐infected cells, in absence of any labelling; however, individual particle types produced by HCMV‐infected cells could not be distinguished based on side scatter alone.

### 3.2 Discrimination and Specific Quantification of HCMV Virions by SYTO 13 Staining Coupled With nFC

We next sought to establish a protocol for specific identification and quantification of HCMV virions within the complex extracellular particle mixtures produced by HCMV‐infected cells. To this end, we sought to exploit the differences in both nucleic acid content and side scatter.

We collected conditioned medium from uninfected and HCMV‐infected HFF cells, and incubated it with increasing concentrations of SYTO 13 nucleic acid stain (100 nM—10 µM). SYTO 13 was chosen due to its previously reported ability to stain the virions of the related herpesvirus herpes simplex virus 1 (HSV‐1) (Loret et al. 2012). SYTO 13 staining resulted in the identification of a distinct population characterised by high side scatter and high fluorescence intensity in the conditioned medium of HCMV‐infected HFF cells (Figure S2A), but not the uninfected cells (Figure S2B). These features were consistent with the predicted HCMV side scatter/fluorescence profile. The identified HCMV population was therefore gated and quantified using a concentration standard (Figure S3A), the median fluorescence intensities recorded (Figure S3B), and the staining index at each SYTO 13 concentration calculated (Figure S3C). This analysis revealed that the measured HCMV virion concentration was consistent across all SYTO 13 concentrations used, except for 100 nM. Similarly, the staining index was indicative of robust separation between the stained HCMV population and the unstained events at all SYTO 13 concentrations except 100 nM. Following this optimisation, 2 µM was chosen for subsequent experiments.

**FIGURE 3:**
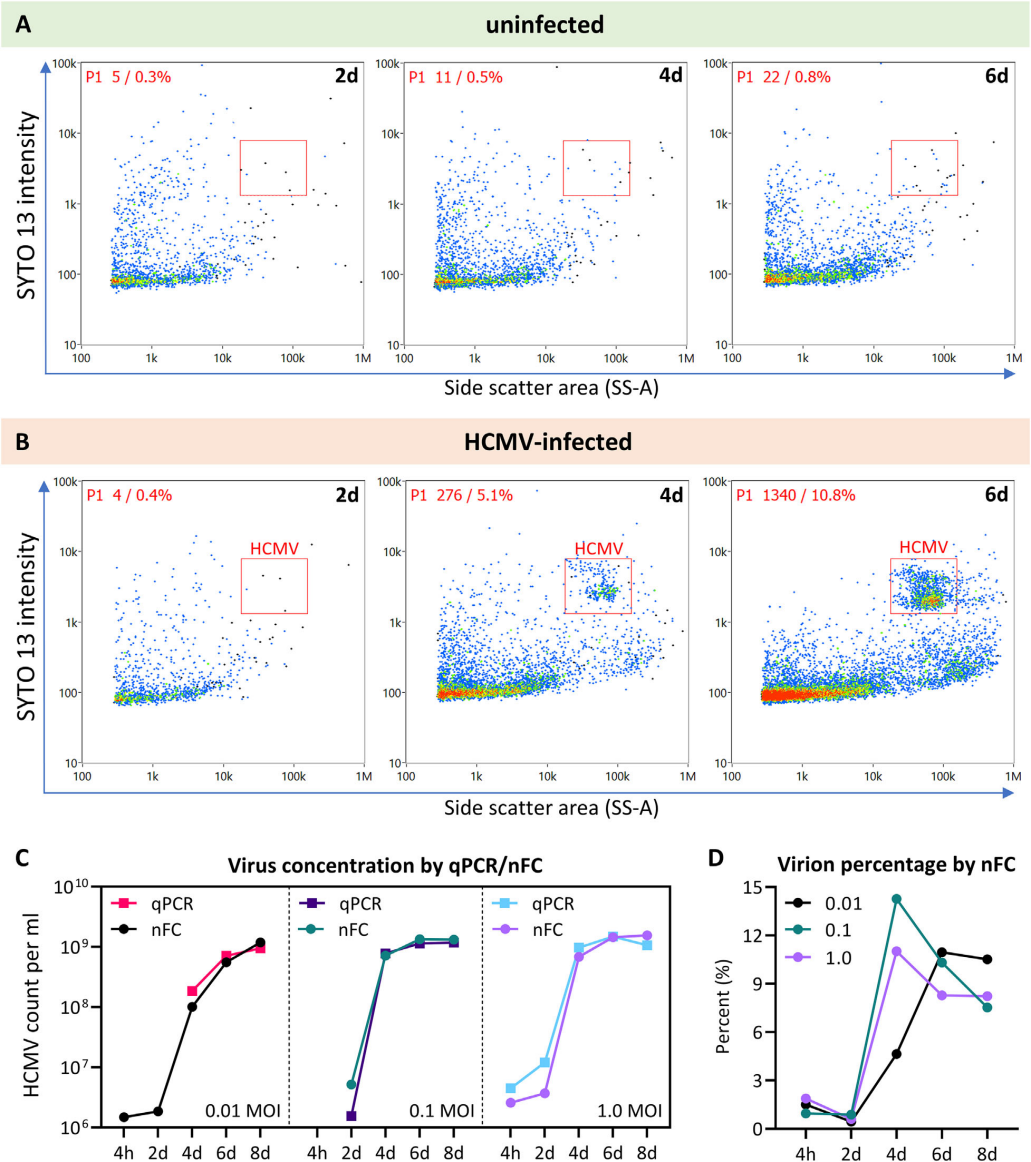
Measurement of HCMV replication kinetics by nFC and qPCR. HFF cells were infected at MOIs of 0.01, 0.1 and 1.0, or left uninfected. Conditioned medium was collected at indicated timepoints, fixed with 4% PFA, incubated with 2 µM SYTO 13 in DPBS, and analysed by nFC by excitation at 488 nm and measurement of emitted light through 488 ± 5 nm (side scatter) and 525 ± 20 nm (SYTO 13) bandpass filters. HCMV virions were identified as a distinct population, gated, and quantified using a concentration standard. The same samples (unfixed) were analysed by qPCR to obtain HCMV genome concentrations (Figure S4). (A and B) Dot plots of SYTO 13‐stained uninfected (A) and HCMV‐infected (B) HFF conditioned medium particles analysed by nFC at 2, 4 and 6 days. HCMV virions were detected as a distinct population (gate shown in red), and their quantity increased over the time course. The HCMV virion population was not observed from uninfected cells. (C) Virion and genome copy number concentrations measured by nFC and qPCR, respectively, were consistent across the timepoints and MOIs tested. (D) Virion percentages across the timepoints and MOIs, as measured by nFC, by gating on the virion population. d = days.

The pre‐optimised SYTO 13 staining conditions were then applied to an HCMV infection time‐course experiment, in which HFF cells were infected with HCMV at MOIs of 0.01, 0.1 and 1.0, for up to 8 days. The HCMV virion populations were again observed in the HCMV‐infected HFF conditioned media (Figure 3B), while no such events were observed in uninfected HFF conditioned media (Figure 3A). To confirm the specificity of the HCMV identification by nFC, the same samples were analysed for viral genome copy number concentration by quantitative PCR, using an internationally certified HCMV standard (Figure S4) (Fryer et al. 2016). Both techniques revealed consistent time‐ and MOI‐dependent increases in HCMV concentration (Figure 3C). The percentage of HCMV virions as measured by nFC was found to be below 15% at all timepoints and MOIs tested (Figure 3D), indicating that abundant non‐virion particles were secreted by the infected cells in parallel.

### 3.3 Simultaneous Quantification of HCMV Virions, Dense Bodies (DBs) and EVs by nFC

In the initial SYTO 13 staining experiments, we observed additional particle populations alongside HCMV virions (Figure 3B). To improve their separation, and decrease fluorescence background levels, we purified extracellular particles from conditioned medium by size exclusion chromatography (SEC). Purified particles were then stained with SYTO 13 (as before), and two other plasma membrane‐permeable fluorogenic dyes with similar excitation/emission profiles, SYBR Green I and SYBR Safe.

**FIGURE 4:**
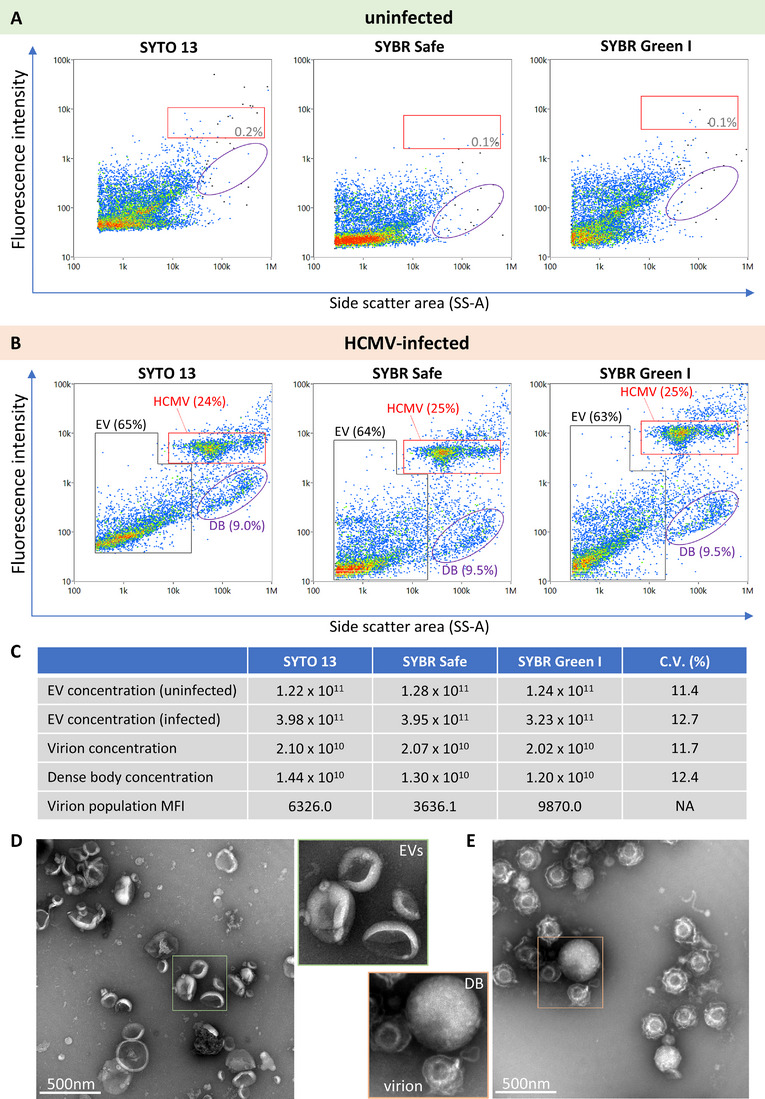
Discrimination of HCMV virions, dense bodies and EVs by nucleic acid staining. Extracellular particles secreted into conditioned media were purified by size exclusion chromatography, fixed with 4% PFA, and stained with SYTO 13, SYBR Safe or SYBR Green I. Samples were analysed by nFC by excitation at 488 nm and measurement of side scatter through the 488 ± 5 nm filter and emitted fluorescence through the 525 ± 20 nm filter. The populations of interest (HCMV virions, EVs and DBs) identified in the dot plots based on their distinct side scatter/fluorescence signatures were gated and quantified using a concentration standard. Control samples are shown in Figure S5. The same samples were visualised by transmission electron microscopy (TEM), to identify the different particle types observed by nFC. (A) Dot plots of uninfected HFF extracellular particles stained with the three nucleic acid dyes. Particles exhibited similar staining patterns with all three dyes, with a proportion of labelled EVs visualised above the unstained background levels. (B) Dot plots of HCMV‐infected HFF extracellular particles stained with the three nucleic acid dyes revealed three major populations: the HCMV virions, the DBs, and the EVs. The HCMV and DB populations were only seen from HCMV‐infected cells. (C) A table summarising mean concentrations of gated HCMV virions, DBs and EVs (*N* = 4), and the virion population MFIs for each of the three dyes. Coefficients of variation (C.V.) are shown for each of the measured parameters (mean, *N* = 4). (D and E) TEM imaging of extracellular particles produced by uninfected (D) and HCMV‐infected (E) HFF cells. EVs were the only particle type observed from uninfected cells, as expected, while large numbers of HCMV virions and DBs were also noted from HCMV‐infected cells.

Analysis by nFC yielded similar results as those from conditioned media, with consistent identification of the HCMV populations only from infected cells (Figure 4B,A). Of the three dyes, SYBR Green I (Figure 4B, right) exhibited the highest median fluorescence intensity (MFI), followed by SYTO 13 (Figure 4B,C). Additionally, lower background fluorescence was observed in SYBR Green I‐ and SYBR Safe‐stained samples compared to SYTO 13. Some virions were found to aggregate, as evidenced by a small number of additional events observed next to and above the main virion population (Figure 4B).

In addition to the virus population, SEC purification facilitated visualisation of additional particle types secreted by HCMV‐infected cells. This included a population characterised by low side scatter intensity and varied degrees of fluorescence, which were identified as EVs (black gates). An additional population with high side scatter and comparably lower fluorescence than HCMV virions was also observed, and these characteristics were consistent with DBs (Figure 4B; purple gates). Importantly, as with the HCMV virions, DBs were not seen from uninfected cells (Figure 4A). To assess the consistency in the measured parameters, we computed coefficients of variation among the three tested dyes. The coefficients of variation all came out to about 12%, indicating that the three dyes yielded consistent results (Figure 4C). Lastly, no appreciable numbers of particles were detected by nFC in various buffer/reagent controls (Figure S5A–D), while unstained controls exhibited no fluorescence and were positioned at the background level (Figure S5E and F).

In order to visually confirm the identity of the populations identified by nFC, we performed transmission electron microscopy on the same SEC‐purified extracellular particles produced by uninfected and HCMV‐infected HFF cells. This confirmed the presence of numerous virions and DBs, but also EVs, from HCMV‐infected cells (Figure 4E), and EVs alone from uninfected cells (Figure 4D), in agreement with the data acquired by nFC.

These analyses confirmed that nucleic acid staining enabled full discrimination of the major extracellular particle types produced by HCMV‐infected cells, wherein each particle type displayed a distinct fluorescence/side scatter profile when analysed by nFC.

### 3.4 Comparison of Virion Sizing by nFC and Transmission Electron Microscopy

Initial nFC experiments reported a mean HCMV virion diameter of about 140 nm (Figure 2E), which is lower than the 150–200 nm reported elsewhere (Seitz et al. 2010). We explored whether inclusion of additional, larger silica nanosphere size standards or changing of side scatter decay settings during acquisition would improve nFC sizing; however, neither of these two modifications had a major impact on the reported virion size (Figure S6).

**FIGURE 5:**
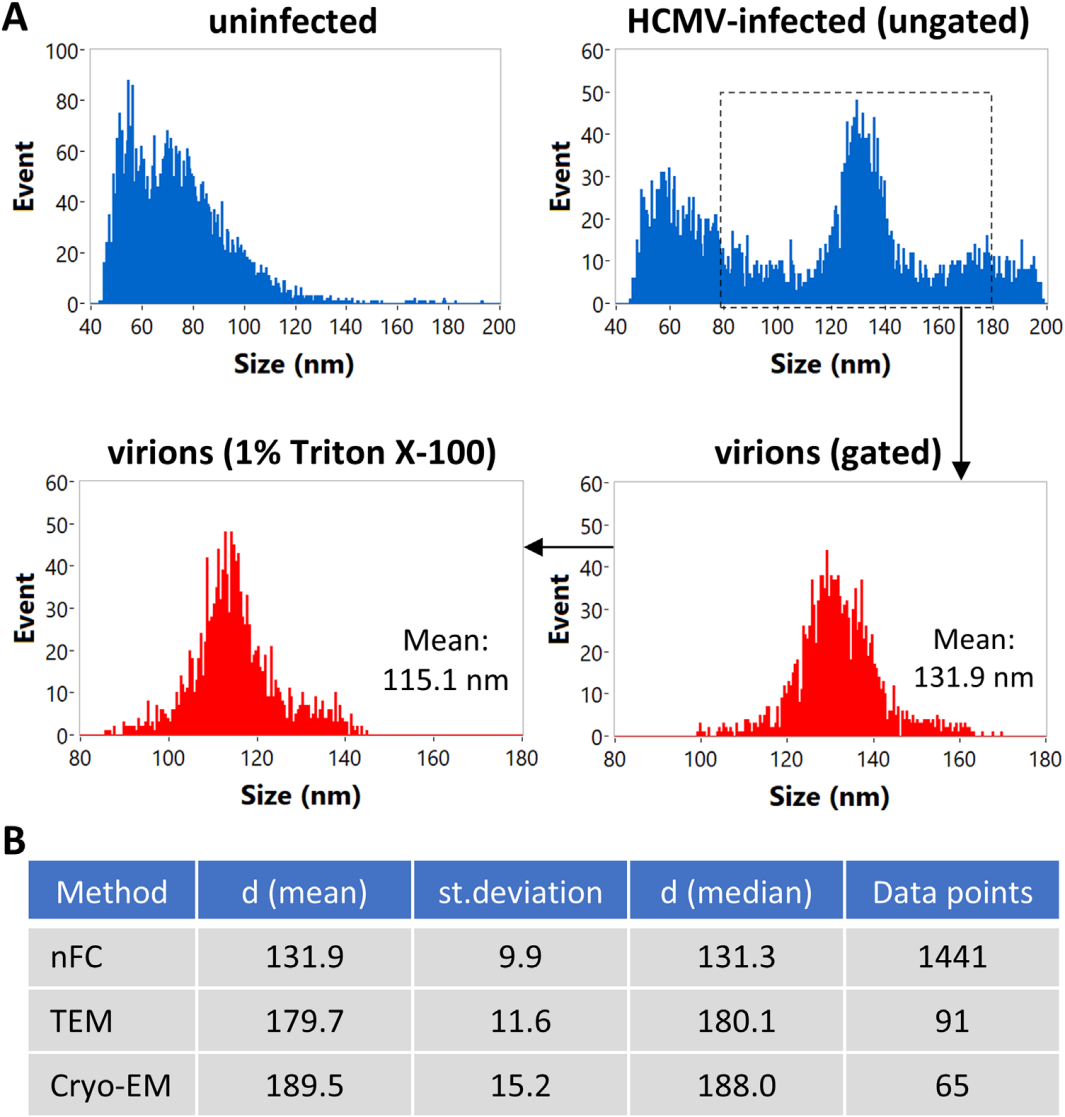
HCMV virion sizing by nFC, TEM and cryo‐EM. Extracellular particles purified by size exclusion chromatography were analysed by nFC and negative stain TEM for virion size. Virion sizing by nFC was performed on untreated virions and those treated with 1% Triton X‐100, which were labelled with SYBR Green I, and gated as before. Additional sizing standards and different side scatter decay settings were also compared (Figure S6). Viral particles prepared by gradient ultracentrifugation were also analysed for virion size by cryo‐EM. Representative micrographs of the HCMV virions included in the sizing analyses by negative stain TEM and cryo‐EM are shown in Figure S7. (A) HCMV virion sizing by nFC. Size distributions were generated for all extracellular particles as well as gated virions only (untreated and treated with Triton X‐100). The mean virion diameter was found to be 131.9 nm, decreasing to 115.1 nm upon Triton X‐100 treatment. Data are representative of three independent experiments. (B) Statistical summary of the HCMV virion sizing results obtained by the three techniques, including mean diameter, standard deviation, median diameter and the number of data points included in the analysis. d = diameter.

In a separate experiment, treatment of HCMV virions with 1% Triton X‐100 brought about a reduction in the reported virion size of 16.8 nm (Figure 5A), indicating that nFC was sensitive to changes in lipid composition. To more accurately assess the size of the HCMV virions, we performed density gradient ultracentrifugation, followed by cryo‐EM (Figure S7). The mean diameter of the HCMV virion analysed by cryo‐EM was found to be 189.5 nm, compared to 131.9 nm by nFC and 179.7 nm by negative stain TEM (Figures 5B and S7). This confirmed that nFC underestimated the size of the HCMV virions.

### 3.5 Separation of Extracellular Vesicles From HCMV Virions and Dense Bodies by High‐Speed Centrifugation Through Density Cushions

**FIGURE 6:**
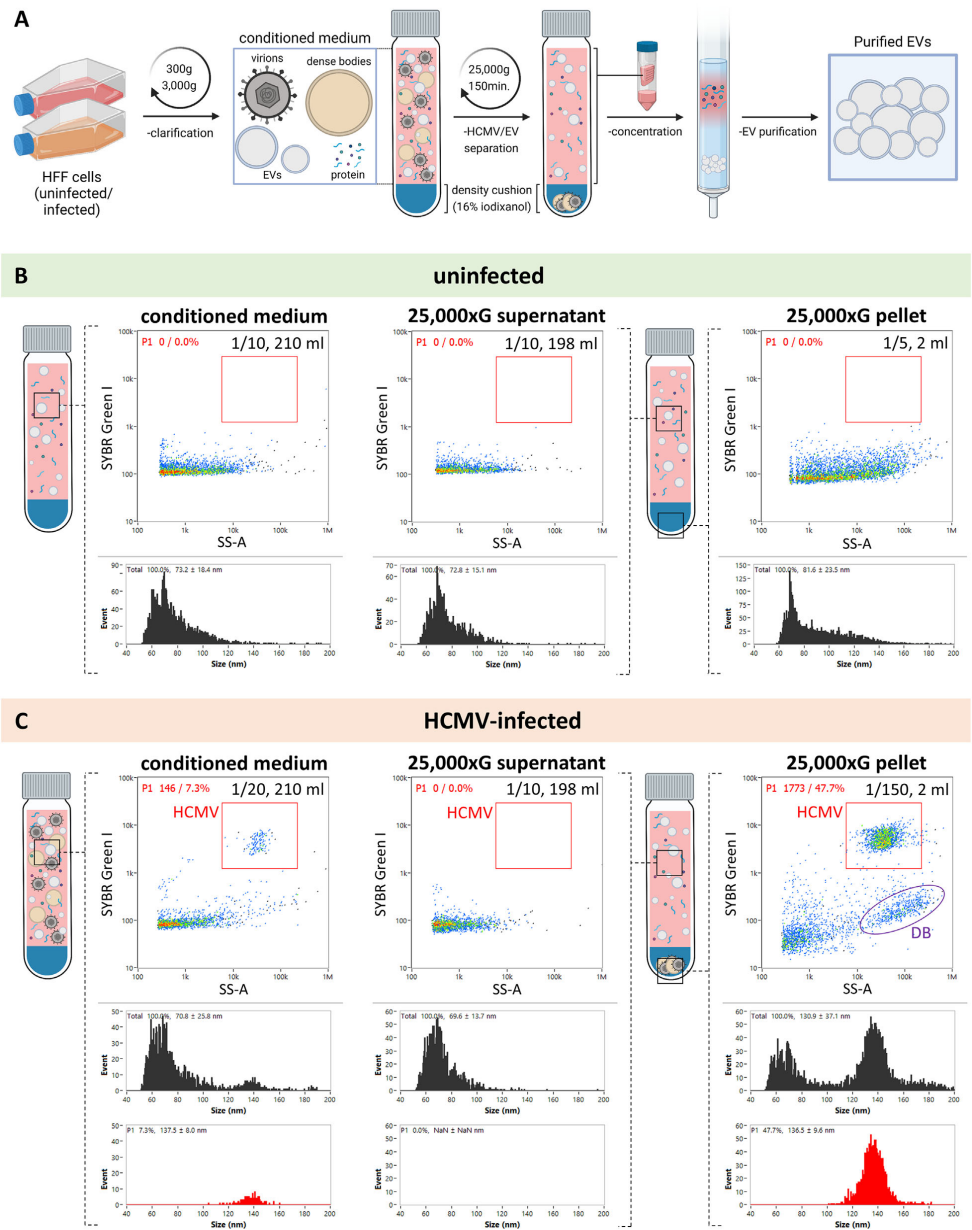
Separation of EVs from viral particles in the HCMV‐infected HFF conditioned medium. Conditioned medium was clarified by centrifugation and 0.45‐µm filtration, and then centrifuged at 25,000 × *g* over underlaid 16% iodixanol cushions. The resulting supernatants and pellets as well as the starting conditioned medium were analysed for particle concentration and size by nFC following SYBR Green I labelling, and by plaque assays for infectivity. (A) Illustration of the methodological approach for the separation of EVs from viral particles and medium contaminants. (B and C) Dot plots and size distributions obtained by nFC following SYBR Green I labelling of uninfected (B) and HCMV‐infected (C) HFF conditioned medium particles, before and after the separation step. Dilutions of samples for the nFC analysis and their total volumes are noted in the top right corner of each dot plot. No major changes were observed between the pre‐ and post‐centrifugation samples in the uninfected HFF samples, with very few particles found in the pellets. In contrast, nFC revealed a total depletion of HCMV virions and DBs from the supernatants, and a concomitant enrichment in the pellets, following the high‐speed centrifugation step. All plots in the figure are representative of four independent experiments. Numerical results are shown in Table 1. Transmission electron microscopy of the 25,000 × *g* pellets is shown in Figure S8. Graphical elements of the figure were created in BioRender. Bokun, V. (2025) https://BioRender.com/d12r423.

Next, we aimed to physically separate HCMV virions and DBs from EVs, by applying our optimised and validated nFC protocols to screen for purity. Due to the appreciably higher density of HCMV virions and DBs, we established a purification strategy utilising high‐speed centrifugation over iodixanol density cushions, designed to be penetrable by the denser HCMV virions and DBs, but not by EVs (Figure 6A).

Using SYBR Green I staining and nFC, we observed no major changes in particles from uninfected HFF conditioned medium before and after high‐speed centrifugation (Figure 6B), with the majority of particles retained in the supernatant (Table 1). In contrast, we observed a complete depletion of viral particles in HCMV‐infected HFF conditioned media following centrifugation, with a concomitant enrichment in the pellets (Figure 6C). As summarised in Table 1, 99.2% of all recovered virions were found in the pellets, and only 0.832% in the supernatant, with a 442‐fold depletion of virions compared to the starting conditioned medium. The pattern of DB depletion was consistent with that of virions, with these particles also no longer observed in supernatants after centrifugation (Figure 6C). We confirmed that the pellets indeed contained HCMV virions and DBs, and not contaminants such as apoptotic bodies or EVs, by TEM (Figure S8). As shown in Figure S8, all particles observed were either DBs or HCMV virions, the latter visualised either as uranyl acetate‐penetrated dark particles or bright circular particles where uranyl acetate had not penetrated to reveal the internal morphology.

**TABLE 1:** A numerical summary of the separation protocol. The total counts of the different particle types, the virion percentages, the virion recovery, and the infectivity in the samples collected during the high‐speed centrifugation protocol are provided. Data are presented as means of four independent experiments, with the range given in parentheses. All samples were analysed in triplicate by both nFC and plaque assay.

	Conditioned medium	25,000 × *g* supernatant	25,000 × *g* pellet
Total particles (uninfected)	5.07 × 10^11^ (3.43 × 10^11^–8.16 × 10^11^)	2.94 × 10^11^ (1.90 × 10^11^–3.73 × 10^11^)	1.29 × 10^10^ (9.26 × 10^7^–3.03 × 10^10^)
Total particles (HCMV‐infected)	3.28 × 10^12^ (1.17 × 10^12^–5.87 × 10^12^)	1.36 × 10^12^ (5.57 × 10^11^–2.96 × 10^12^)	2.75 × 10^11^ (1.06 × 10^11^–6.67 × 10^11^)
Total HCMV virions	2.79 × 10^11^ (9.30 × 10^10^–4.62 × 10^11^)	1.06 × 10^9^ (9.24 × 10^7^–1.93 × 10^9^)	1.33 × 10^11^ (5.33 × 10^10^–2.88 × 10^11^)
Virion percent of all particles	8.59% (7.87%–9.88%)	0.0781% (0.0154%–0.128%)	52.0% (43.2%–59.5%)
Percent of total virion recovery	Starting material (100%)	0.832% (0.173%–1.74%)	99.2% (98.3%–99.8%)
Infectivity (PFU/mL)	1.19 × 10^6^ (2.27 × 10^5^–3.80 × 10^6^)	4.00 × 10^2^ (0–1.27 × 10^3^)	3.77 × 10^6^ (7.00 × 10^5^–7.20 × 10^6^)
Particle:PFU ratio	2968 (589–6395)	NA	11,368 (8305–19,681)

In line with nFC data, HCMV infectivity as measured by plaque assays was 1549‐fold depleted in the supernatants compared to the starting conditioned medium (Table 1). To investigate infectivity in a normalised fashion, we compared particle:PFU ratios in the conditioned medium and the pellets, as an indication of the functionality of the virus. Particle:PFU ratio averaged 2968 in the conditioned medium and 11,368 in the pellets, comprising a 3.83‐fold change, suggesting that there was a limited effect of the separation protocol on the normalised viral infectivity (Table 1).

In summary, high‐speed centrifugation through 16% iodixanol cushions proved to be an effective and simple approach for separating EVs from HCMV virions and DBs, based on their physical differences, while preserving infectivity of the isolated virus.

### 3.6 Purification of EVs From Virion‐ and Dense Body‐Depleted Supernatants by Size Exclusion Chromatography

Following depletion of virions and dense bodies from HCMV‐infected cell conditioned medium by high‐speed centrifugation, we combined ultrafiltration and SEC to purify EVs from the remaining medium contaminants.

**FIGURE 7:**
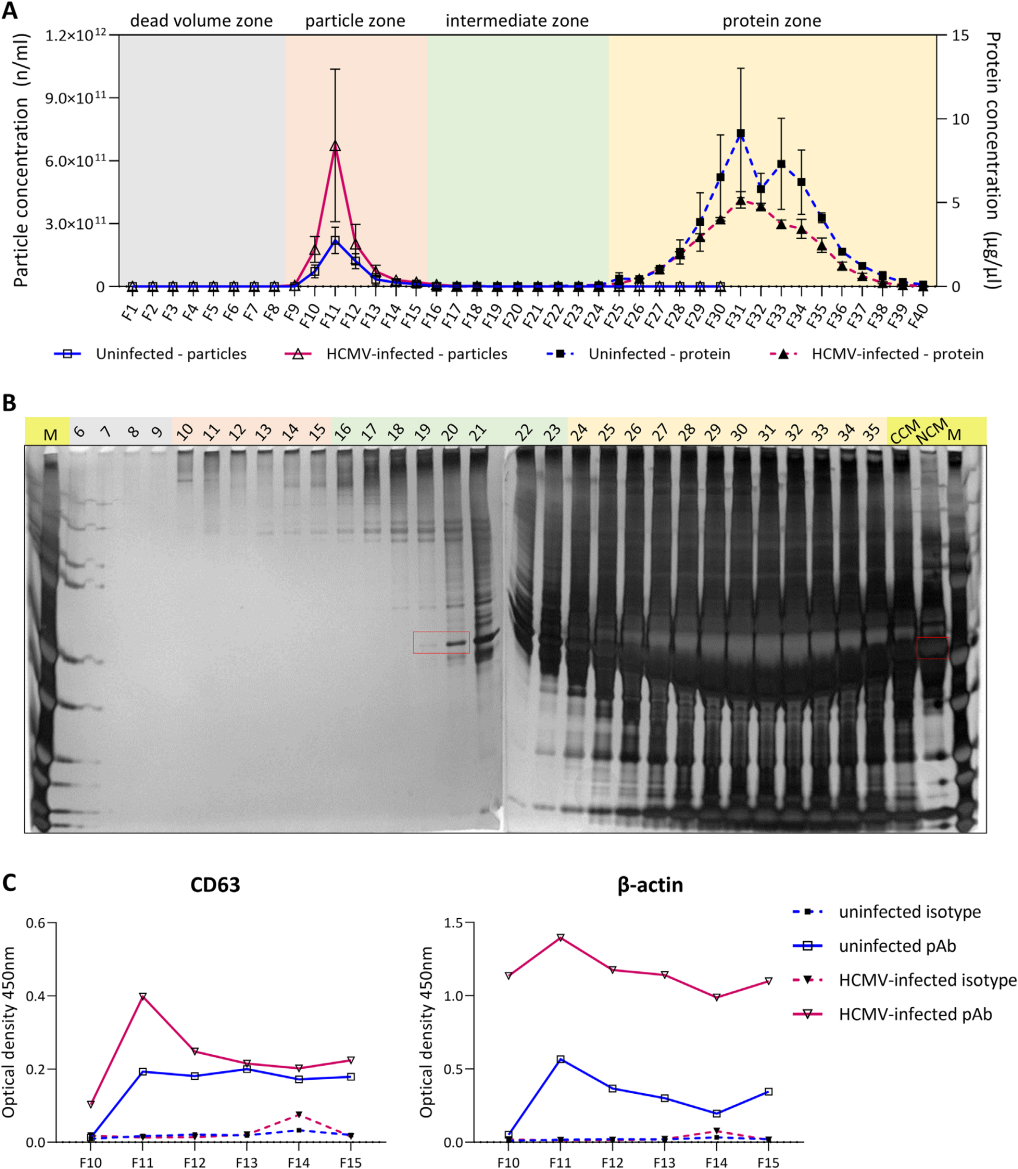
Size exclusion chromatography purification of EVs from post‐25,000 × *g* centrifugation supernatants. EVs remaining in the supernatants following high‐speed centrifugation were concentrated by 100‐kDa ultrafiltration, and fractionated on a custom Sepharose CL‐2B column. (A) EV/protein elution profiles as measured by nanoparticle tracking analysis (NTA) and Bradford assay show a robust separation of EVs from the contaminating protein. (B) Silver staining of fractions 6–35 from the HCMV‐infected cells, including the starting conditioned medium (CCM), and the non‐conditioned medium (NCM). Bovine serum albumin (BSA), the most abundant component of the fetal bovine serum (FBS) in the complete growth medium, appeared in fraction 19 and peaked in fraction 31 (indicated with red rectangles). (C) ELISA for CD63 and β‐actin shows positivity for these two EV‐associated proteins in the particle‐containing fractions. Matching NTA and Bradford assay data obtained from non‐conditioned complete medium is shown in Figure S9A while the matching silver staining data for uninfected cells is shown in Figure S9B. Data in panel A are presented as means ± SEMs (*N* = 3), with all measurements performed in triplicate. Data included in panels B and C are from single experiments. ELISA measurements were performed in duplicate. F = fraction; M = molecular weight markers.

When the supernatants were concentrated by ultrafiltration and applied to a custom Sepharose CL‐2B SEC column, EVs eluted in fractions 10–15 and free protein in fractions 23–40, as evidenced by nanoparticle tracking analysis and Bradford assay, respectively (Figure 7A). Identical processing and analysis of non‐conditioned complete medium confirmed absence of contaminant particles (Figure S9A). Using silver staining, we confirmed that contaminating soluble proteins (such as BSA; marked with red rectangles) eluted in later fractions for both HCMV‐infected (Figure 7B) and uninfected cells (Figure S9B). The total average yield of EVs from three independent experiments was found to be 1.43 × 10^12^ for uninfected and 3.53 × 10^12^ for HCMV‐infected cells.

**FIGURE 8:**
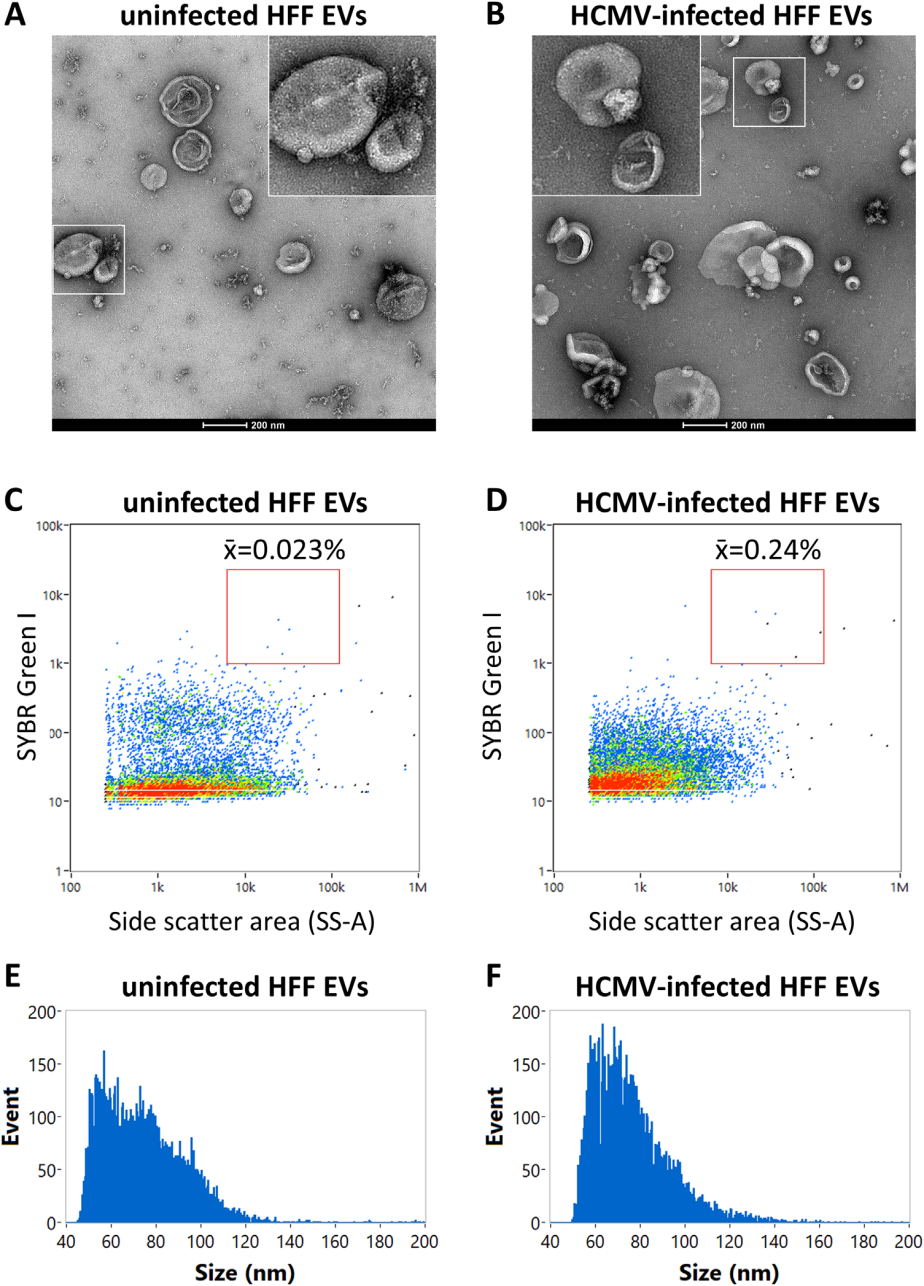
Characterisation of uninfected and HCMV‐infected HFF EVs. EVs were purified from 25,000 × *g* supernatants by SEC and concentrated by ultrafiltration. EVs were examined by TEM and nFC. (A and B) TEM imaging of HFF EVs revealing characteristic circular/oval EV morphology. No HCMV virions were observed in EV preparations from infected cells. Additional widefield micrographs are included in Figure S10. (C and D) Representative nFC dot plots showing SYBR Green I labelling of uninfected and HCMV‐infected HFF EVs. The mean percentage of events in the virion gate is indicated above. (E and F) Representative size distributions of purified HFF EVs generated by nFC. The data in panels (A) and (B) are representative of two independent experiments, and the data in panels (C–F) are representative of five biological replicates.

Fractions 10–15 were positive for CD63 and β‐actin (Figure 7C) when analysed by a solid phase binding assay, confirming the identity of EVs in these fractions. The EVs from both uninfected and infected cells also displayed characteristic EV morphology by TEM, with no obvious morphological differences between the two (Figures 8A,B and S10). While we did not observe any virions or DBs in infected HFF EV preparations by TEM (Figure 8B), near‐undetectable numbers of virions were noted in some of the SYBR Green I‐stained preparations by nFC (Figure 8C,D). Lastly, purified EVs produced by both uninfected and infected cells were positive for the EV surface marker CD81 by nFC (Figure S11). EVs from uninfected cells exhibited 23% positivity (Figure S11A), while those from infected cells had a positivity of 38.5% (Figure S11B).

## 4 Discussion

The potential involvement of EVs in viral infection is increasingly appreciated, but co‐investigation of EVs and viruses has been challenging due to the similarities in their biophysical characteristics (Alenquer and Amorim 2015; Sadeghipour and Mathias 2017; Anderson et al. 2016; Hoen et al. 2016; Zhou and McNamara 2020; Metzner and Zaruba 2021; McNamara and Dittmer 2020). Most virology studies utilise virus preparations also containing EVs, whose parallel effects in various contexts are not considered. Conversely, EV preparations from virus‐infected cells can contain contaminating virions, which could lead to misunderstandings about their functional properties and cargo. This study addresses two major challenges: first, how to efficiently analyse heterogenous extracellular particle mixtures produced during HCMV infection; and second, how to separate viral particles and EVs in complex particle mixtures for downstream experiments or end‐point analyses. We report the applicability of nFC to discriminate and quantify HCMV virions and co‐produced EVs and DBs. Using this as a monitoring tool, we also report an optimised protocol for the separation of these particle types.

The central protocol in this study involved employment of a fluorescent nucleic acid stain combined with nFC, to take advantage of the differential nucleic acid contents and particle sizes for simultaneous discrimination of all major particle types produced by HCMV‐infected cells. All dyes tested here readily penetrated the HCMV nucleocapsids and EVs, to interact with DNA and/or RNA, and exhibited bright fluorescence, meaning that no washing steps were required. In addition to the three dyes reported here (SYTO 13, SYBR Safe I, SYBR Green), similar results were also obtained with SYTO 16 and SYTO 59 (data not shown). While the initial focus was to provide a method to distinguish only HCMV virions, it was apparent that nucleic acid staining also enabled discrimination of all the major particle types produced by HCMV‐infected cells. This included EVs, a proportion of which displayed bright fluorescence, indicating the presence of an abundant DNA and/or RNA cargo. This is consistent with a previous report, which showed that EVs contained abundant double‐stranded DNA, both on the EV surface and inside the lumen (Liu et al. 2022). Using standards, the authors estimated the size of the DNA cargo to be up to about 50,000 bp at most, and rarely above that. This appears to be in line with our data, where the fluorescence of EVs was comparably lower than that of the HCMV virions, which contain a 230,000‐bp DNA genome. Because we employed SYBR Green I, which appears to interact exclusively with DNA, our work reinforces the previous report that EVs do indeed carry DNA as part of their cargo (Liu et al. 2022).

In addition to the speed of the protocol, a major benefit of the nFC protocol highlighted here was the ability to measure particle concentration from very small volumes of crude medium (less than 1 µL). These two benefits position nFC as an attractive tool for near‐real‐time monitoring of viral replication and EV production kinetics. When compared to the more laborious and time‐consuming qPCR, nFC exhibited consistent virion concentrations at each MOI and timepoint tested, further highlighting the accuracy and robustness of the nFC method. The only disadvantage exhibited by nFC was its reporting of smaller virion size compared to the gold‐standard method of cryo‐EM. We believe that this stems from not the technique itself but from calibration materials (silica nanospheres), which possess different light properties—notably the refractive index—compared to HCMV virions. In such circumstances, sizing correction can be applied based on Mie theory (Welsh et al. 2023); however, this requires input of the virion refractive index at the excitation wavelength, the former of which is currently unknown. Future improvements in calibration materials as well as inclusion of sizing correction algorithms in the software accompanying nFC instruments are warranted.

As an additional potential application of nFC relevant to virological research, nFC data was integrated with that generated by viral plaque assays. This allowed us to examine infectivity as normalised to the number of particles present in the sample, and to conclude that our EV purification protocol did not cause significant damage to the co‐isolated viral particles. Therefore, calculation of particle:PFU ratios can be applied to the optimisation of protocols for isolation of infectious virus, or for assessing packaging of mutant, deficient particles that cannot establish infection, a phenomenon observed in many virus families (Hutchinson et al. 2010; Sun et al. 2019; Yoshida et al. 2018; Vignuzzi and López 2019; Topilko and Michelson 1994; Heilingloh and Krawczyk 2017), as well as to assess infectivity of clinical virus strains. Finally, in terms of evaluating the contamination of EV preparations with viral particles, nFC is oblivious to the functionality of the virus, and therefore represents a superior method for this application compared to plaque assays, especially when EV isolation methods that damage virions are employed.

While the protocol employed for specific quantification of HCMV virions described in our work was robust, there are several scenarios in which the same end‐goal would be difficult to achieve for other viruses. The first would be in cases where nucleic acid dyes would not penetrate inside the viral nucleocapsid to interact with the nucleic acid cargo, as reported before (Brussaard 2004), or if the nucleic acid cargo was not substantial enough to produce sufficient fluorescence. The second would be if viral particles were of significantly smaller size and therefore reminiscent of EVs in this regard. Potential solutions to these complications include labelling of one or more viral proteins using antibodies, or labelling other biomolecules such as glycans and/or lipids, or staining the viral genetic material using biorthogonal strategies. It is expected therefore that nFC protocols would have to be optimised based on the properties of different viruses and samples under study.

There are previous works describing the use of flow cytometry for the detection and analysis of viruses and EVs by flow cytometry (Welsh et al. 2023; Kuiper et al. 2021; Lippe 2018; Zamora and Aguilar 2018). The limitations and the unique challenges of EV flow cytometry as well as a framework for methodology reporting have been outlined by Welsh et al (Welsh et al. 2020). Compared to conventional flow cytometers, the nanoFCM flow nanoanalyser utilised in this study incorporates key design adaptations, including reductions of the sample stream diameter and flow rate, and the use of highly sensitive single photon counting modules, which prevent coincident particle detection and improve sensitivity, respectively (Buntsma et al. 2023). As a result of the optimised instrument design and staining protocol, we were able to detect three distinct particle populations—HCMV virions, DBs and EVs—and simultaneously quantify them, which has not been achieved before. In the only previous example of HCMV analysis by flow cytometry, HCMV virions were successfully stained with SYTO 41, and gated based on their high fluorescence, but DBs and potential EVs were not detected as discrete populations, as they were in our experiments (Vlasak et al. 2016). Of note, the authors estimated that the virion size had to be at least 150 nm to be detected by light scatter, in line with another flow cytometer study (Buntsma et al. 2023), whereas nFC enabled us to detect particles down to less than 50 nm.

Finally, utilising our optimised and validated nFC protocol, we established a method for the physical separation of HCMV virions and DBs from EVs, based on high‐speed centrifugation through density cushions. In contrast to previous methods employing ultracentrifugation of density gradients (Turner et al. 2020; Zicari et al. 2018; Bergamelli et al. 2021), our protocol is simpler, takes less time, and uses greatly reduced centrifugation speed and time, thereby minimising potential physical stress of the isolated virions and EVs. While the focus of the experiment was to isolate pure EVs, functional virus was co‐isolated and could be used for experiments or separated from DBs by additional steps. In contrast to wild‐type HCMV strains, the strain utilised in this study, Merlin R1111, replicates productively in fibroblast cells, with a high resulting quantity of produced extracellular virions (Stanton et al. 2010). This, coupled with utilisation of conditioned medium in late stages of infection, meant that our EV purification approach was shown to be robust at separating EVs from large amounts of virus. EVs purified using this protocol were characterised guided by MISEV2018 (Théry et al. 2018) and would be suitable for either functional or biochemical study.

nFC has been previously utilised for the analysis of EVs (Tian et al. 2020), bacteriophages, viral vaccine products, and an adenoviral vector (Ma et al. 2016; Niu et al. 2021). These lower complexity products are different to the complex EV/virus mixtures produced by infected mammalian cells reported here. Our study represents the first application of nFC for systematic examination of a clinically relevant herpesvirus, which infects a significant proportion of the worldwide population (Seitz et al. 2010), with critical consideration given also to the co‐produced EVs and DBs. Additionally, our study is the first study to apply nFC to an informed design of a method for purification of EVs from virally infected mammalian cells, leading to an establishment of a simple and widely applicable protocol for accomplishing this difficult task. Thus, our work represents an advancement and a novel addition to the existing limited literature dealing with the application of nFC to the study of viruses and EVs.

In summary, the work presented here can be used as a framework for the analysis and physical separation of enveloped viruses and EVs. These methods can facilitate investigation of poorly understood areas such as the relationship between virus and EV production, accurate comparisons of virus and EV cargo, and the functional roles of EVs in viral infection.

## Author Contributions

**Vladimir Bokun**: conceptualization (lead), formal analysis (lead), funding acquisition (supporting), investigation (lead), methodology (lead), visualization (lead), writing–original draft (lead), writing–review and editing (equal). **Blair L. Strang**: funding acquisition(Supporting), writing–review and editing(Equal). **Paschalia Pantazi**: methodology (supporting), writing–review and editing (equal). **Yan Liu**: funding acquisition (equal), supervision (supporting), writing–review and editing (equal). **Beth Holder**: Conceptualization (equal), funding acquisition (lead), supervision (lead), writing–review and editing (lead).

## Conflicts of Interest

The authors declare no conflicts of interest.

## Supporting information



Supporting Information

Supporting Information

Supporting Information
