# Supplementary material for: Nano‐Flow Cytometry‐Guided Discrimination and Separation of Human Cytomegalovirus Virions and Extracellular Vesicles
Source: J Extracell Vesicles. 2025 May 2;14(5):e70060. doi: 10.1002/jev2.70060 (PMC12046292; doi:10.1002/jev2.70060)
Supplement: Supplementary file 1 — Supporting Information [file JEV2-14-e70060-s002.pdf]

Supplementary Figure 1

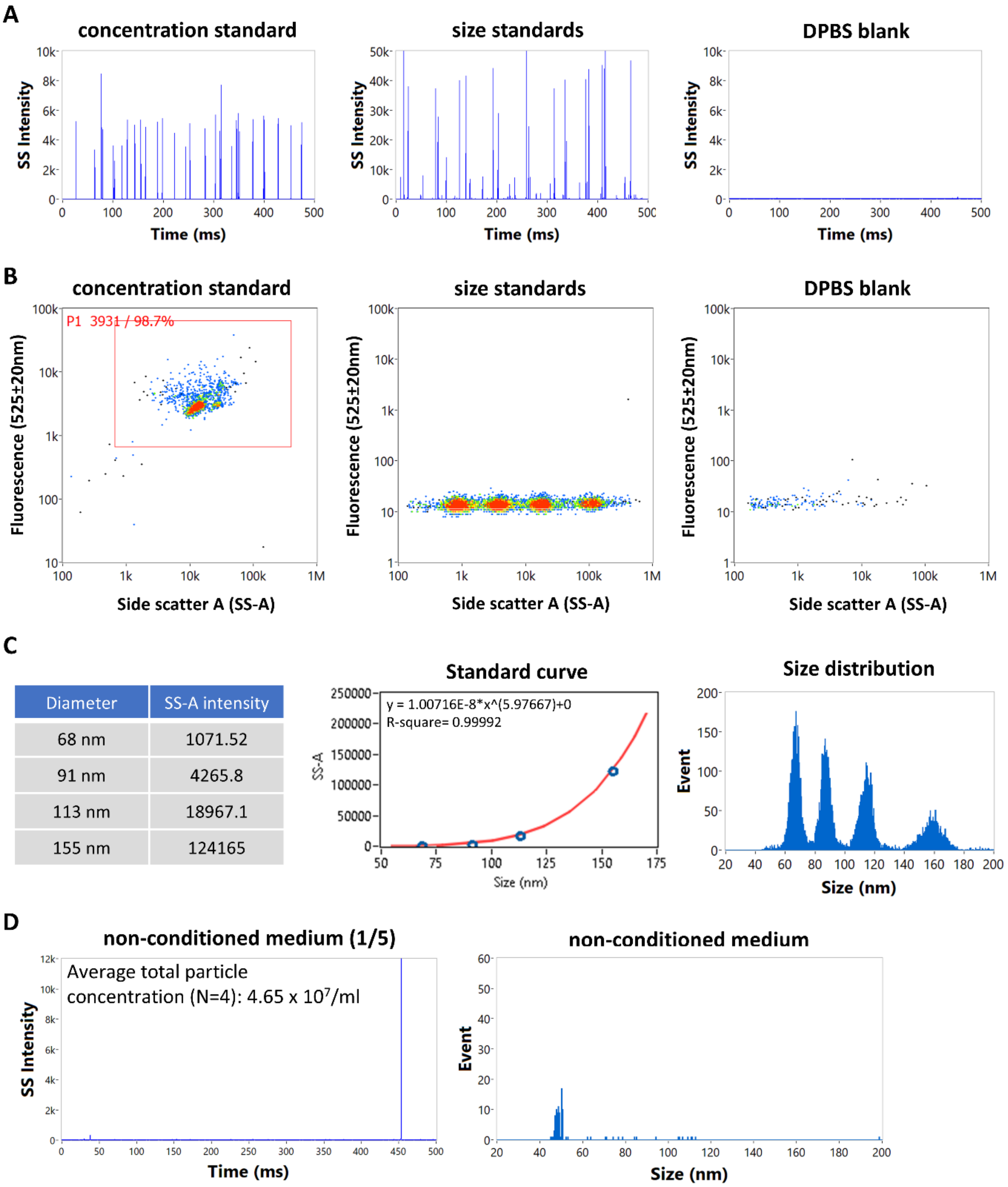

Figure S1. Controls used in nFC experiments.

A. Side scatter burst trace plots from concentration and sizing silica nanosphere standards and a DPBS blank.

B. Dot plots corresponding to the concentration nanosphere standard (left), the sizing nanosphere standards (middle), and a DPBS blank sample (right).

C. Sizes (diameter, in nanometers) and side scatter intensities (A=area) of silica nanosphere sizing standards used for the generation of a standard curve for determination of experimental particle size.

D. A representative burst trace plot and a size distribution corresponding to a non-conditioned complete medium control. Dilution is indicated above the burst trace plot.

Supplementary Figure 2

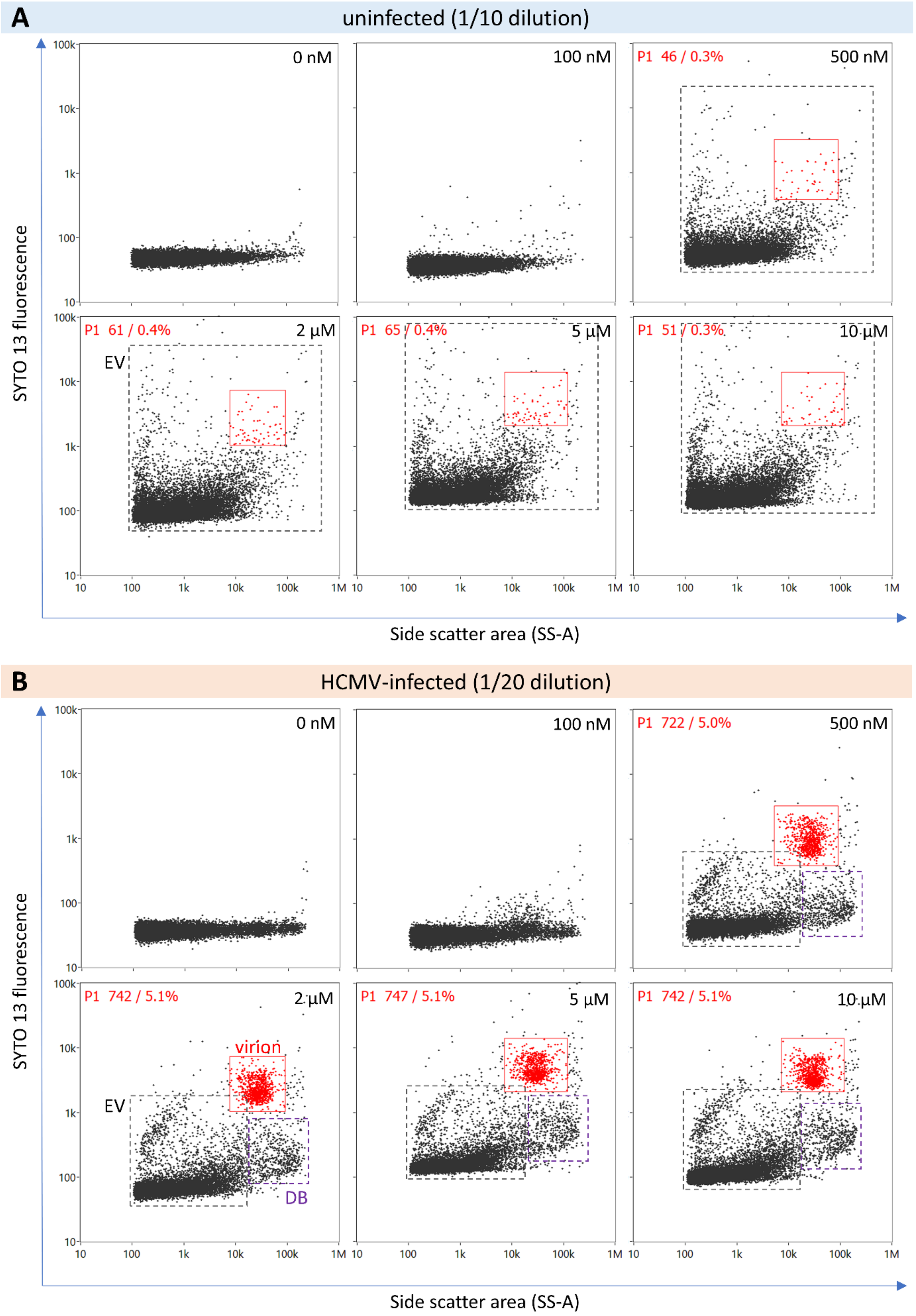

Figure S2. Titration of SYTO 13 concentration for staining of extracellular particles produced by uninfected and HCMV-infected HFF cells.

A. Dot plots showing extracellular particles in the conditioned media from uninfected HFF cells stained with increasing concentrations of SYTO 13.

B. Dot plots showing extracellular particles in the conditioned media from HCMV-infected HFF cells stained with increasing concentrations of SYTO 13.

Sample dilutions are indicated in the parentheses. The concentration of SYTO 13 is indicated in the upper right corner of each dot plot. Data are representative of 2 biological replicates.

Supplementary Figure 3

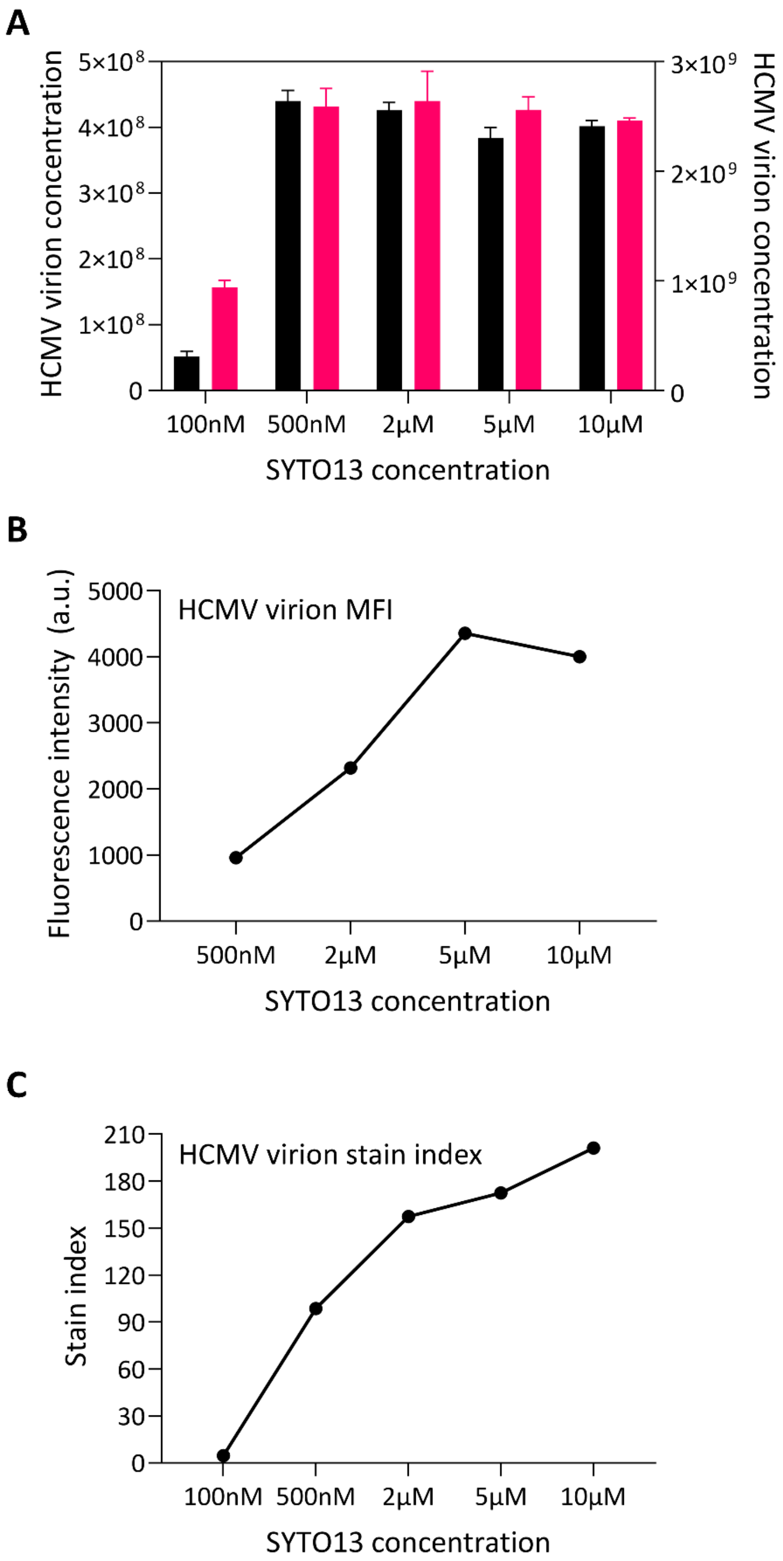

Figure S3. Staining of HCMV virions with different concentrations of SYTO 13. Virions identified in Figure S2 were gated and their concentration determined using a concentration standard, which consisted of silica nanospheres of a known concentration. Median fluorescence intensities were recorded, and the stain index calculated.

A. Concentrations of HCMV virions in two biological replicates as measured by nFC following SYTO 13 staining at different concentrations. The left Y axis is used for the biological replicate shown in black bars; the right Y axis is used for the biological replicate shown in pink bars. The measured concentrations were consistent for both biological replicates at each SYTO 13 concentration, except at 100 nM, which yielded very weak staining.

B. HCMV virion median fluorescence intensities (MFIs) at each SYTO 13 concentration. The MFI increased with increasing SYTO 13 concentration up to 5 µM, then decreased at 10 µM.

C. The HCMV virion stain index at each SYTO 13 concentration. 100 nM was not included due to the weak staining of the virions. The stain index was calculated as described in the methods section. The stain index increased with increasing SYTO 13 concentration.

Supplementary Figure 4

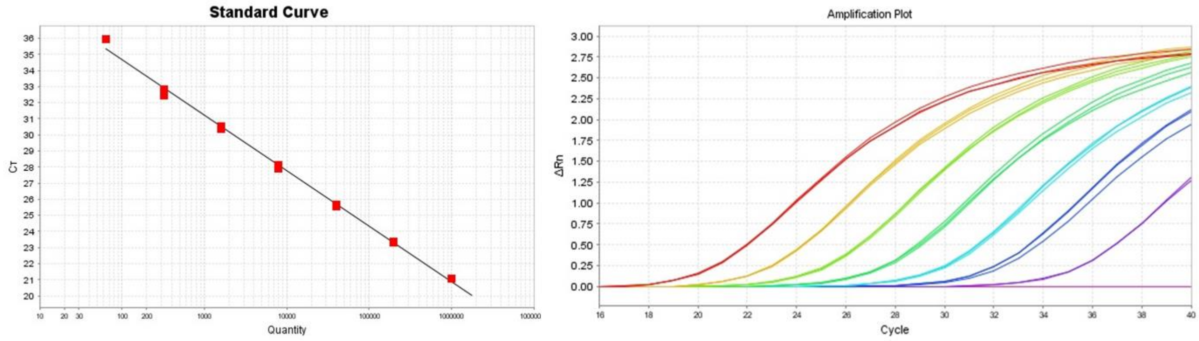

Target: CMV UL132; **Slope:** -3.433; **Y-inter:** 41.556; **R^2:** 0.997; **Eff%:** 95.18

Figure S4. The standard curve used for the quantification of HCMV genome copy numbers by qPCR.

Supplementary Figure 5

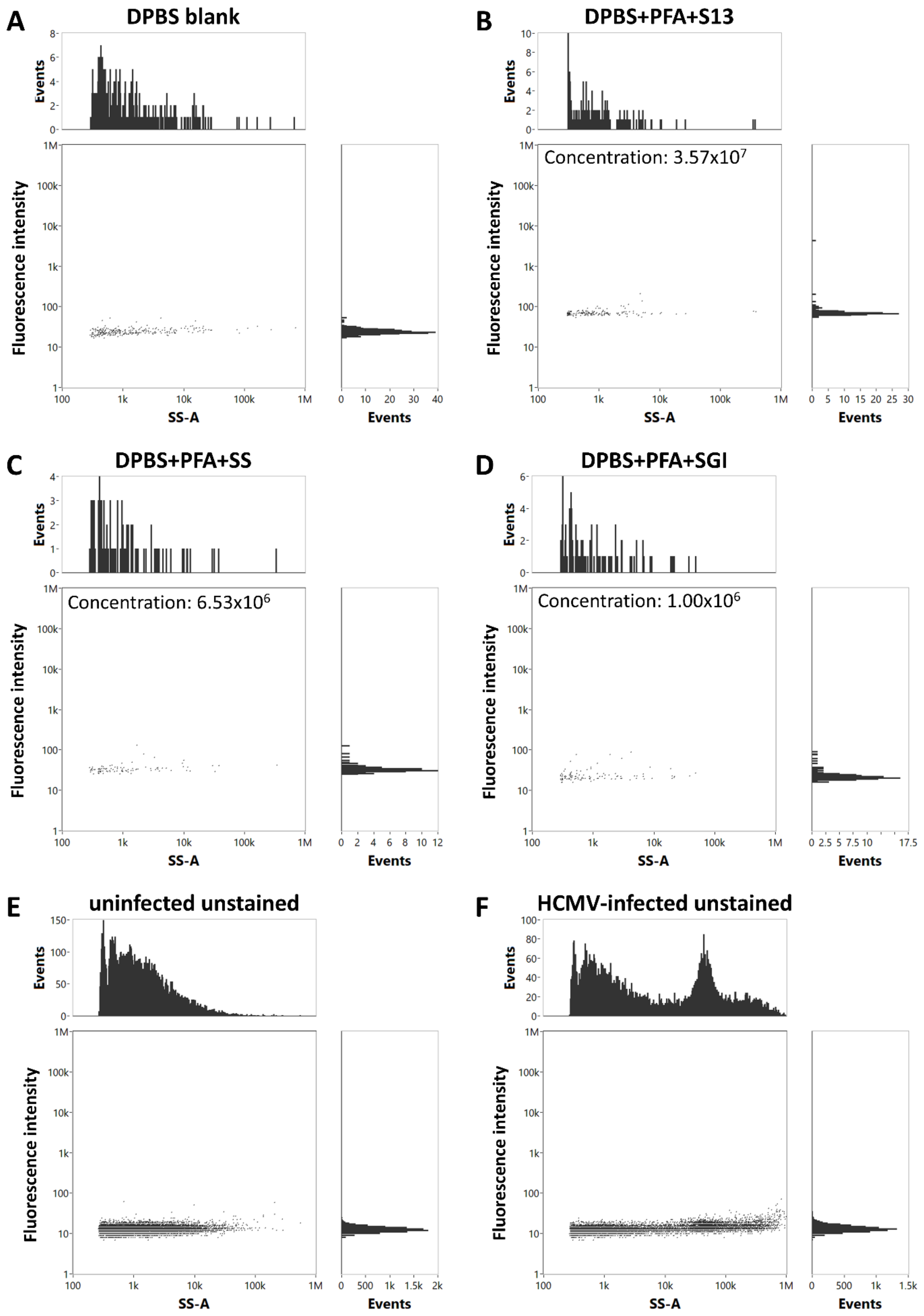

Figure S5. Quality control samples used in the nucleic acid staining experiments.

Included are dot plots corresponding to the blank sample, the dye-only controls, and the unstained experimental sample controls.

A. DPBS blank.

B. DPBS blank with PFA fixative and SYTO 13 (S13; at 2 µM).

C. DPBS blank with PFA fixative and SYBR Safe (SS; at 1X).

D. DBPS blank with PFA fixative and SYBR Green I (SGI; at 1X).

E. Unstained uninfected HFF extracellular particle control.

F. Unstained HCMV-infected HFF extracellular particle control.

Supplementary Figure 6

A

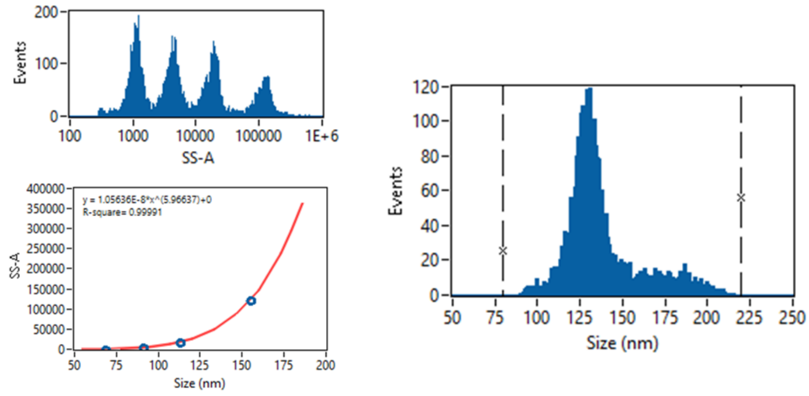

SS decay	10%
Events	5392
Median	131.75
Mean	137.13
SD	20.76

B

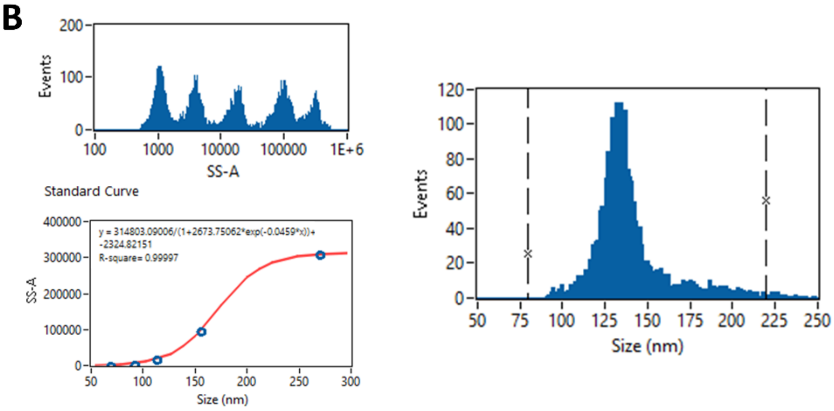

SS decay	10%
Events	4975
Median	133.75
Mean	137.60
SD	19.13

C

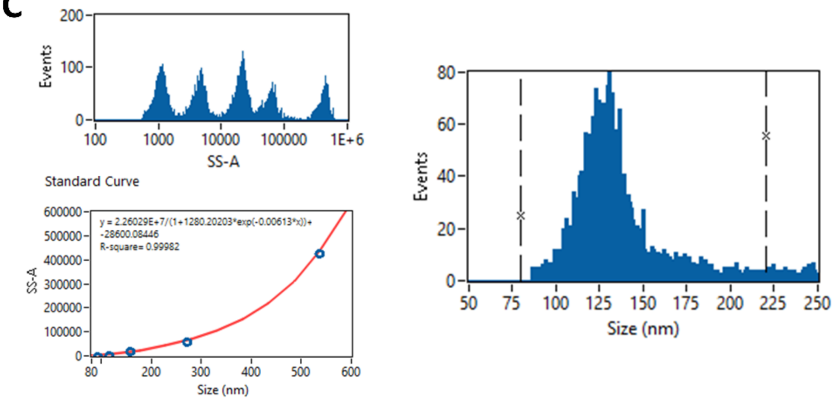

SS decay	2%
Events	4581
Median	129.25
Mean	133.03
SD	22.67

D

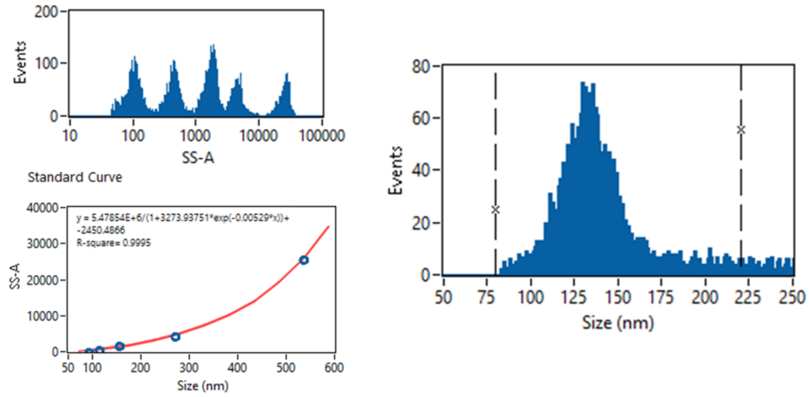

SS decay	0.2%
Events	4728
Median	134.25
Mean	137.73
SD	23.55

Figure S6. The effect of technical parameters on the reported HCMV virion size.

HCMV-infected HFF extracellular particles were purified by size exclusion chromatography and labelled with 2 µM SYTO 13. The same sample was analyzed under three different side scatter decay settings and with additional larger silica nanosphere standards. Size distributions and the mean and median diameters of the gated virion populations were generated via standard curves prepared from silica nanosphere sizing standards acquired at the same settings.

A. Virion sizing under standard settings used throughout the study.

B. Virion sizing under standard settings with an additional 250-nm sizing standard.

C. Virion sizing under 2% side scatter decay setting, without the smallest sizing standard (68 nm) and with two additional larger standards (270 nm and 535 nm).

D. Virion sizing under 0.2% side scatter decay setting without the smallest standard (68 nm) and with two additional larger standards (270 nm and 535 nm).

Supplementary Figure 7

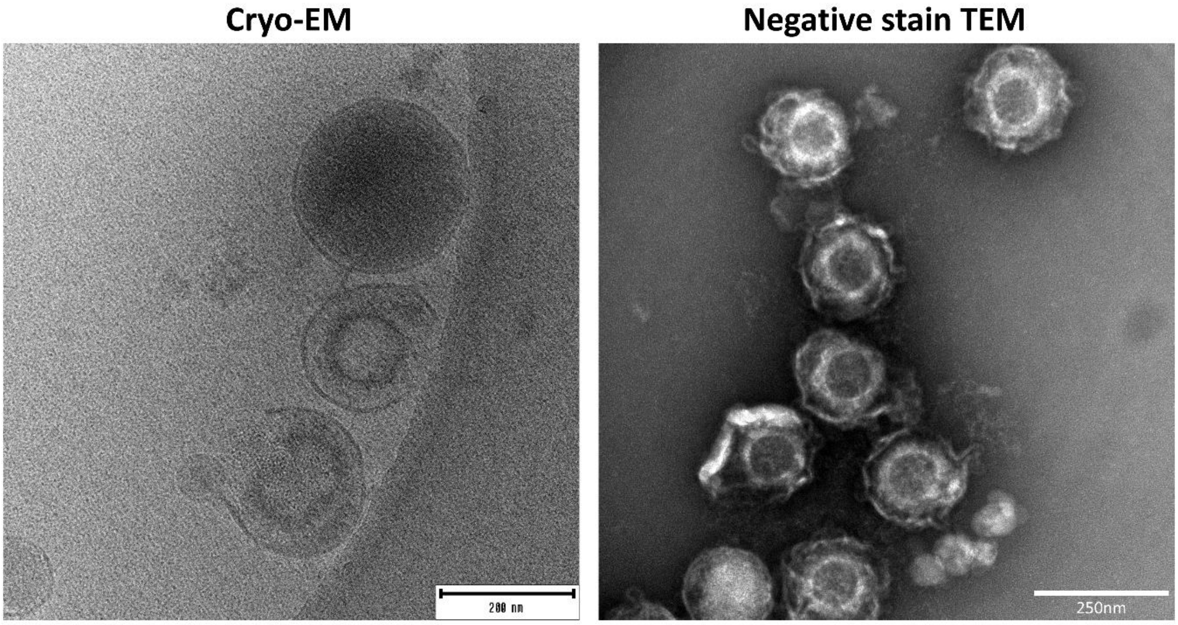

Figure S7. Transmission electron microscopy micrographs of viral particles included in the sizing analyses.

Supplementary Figure 8

A

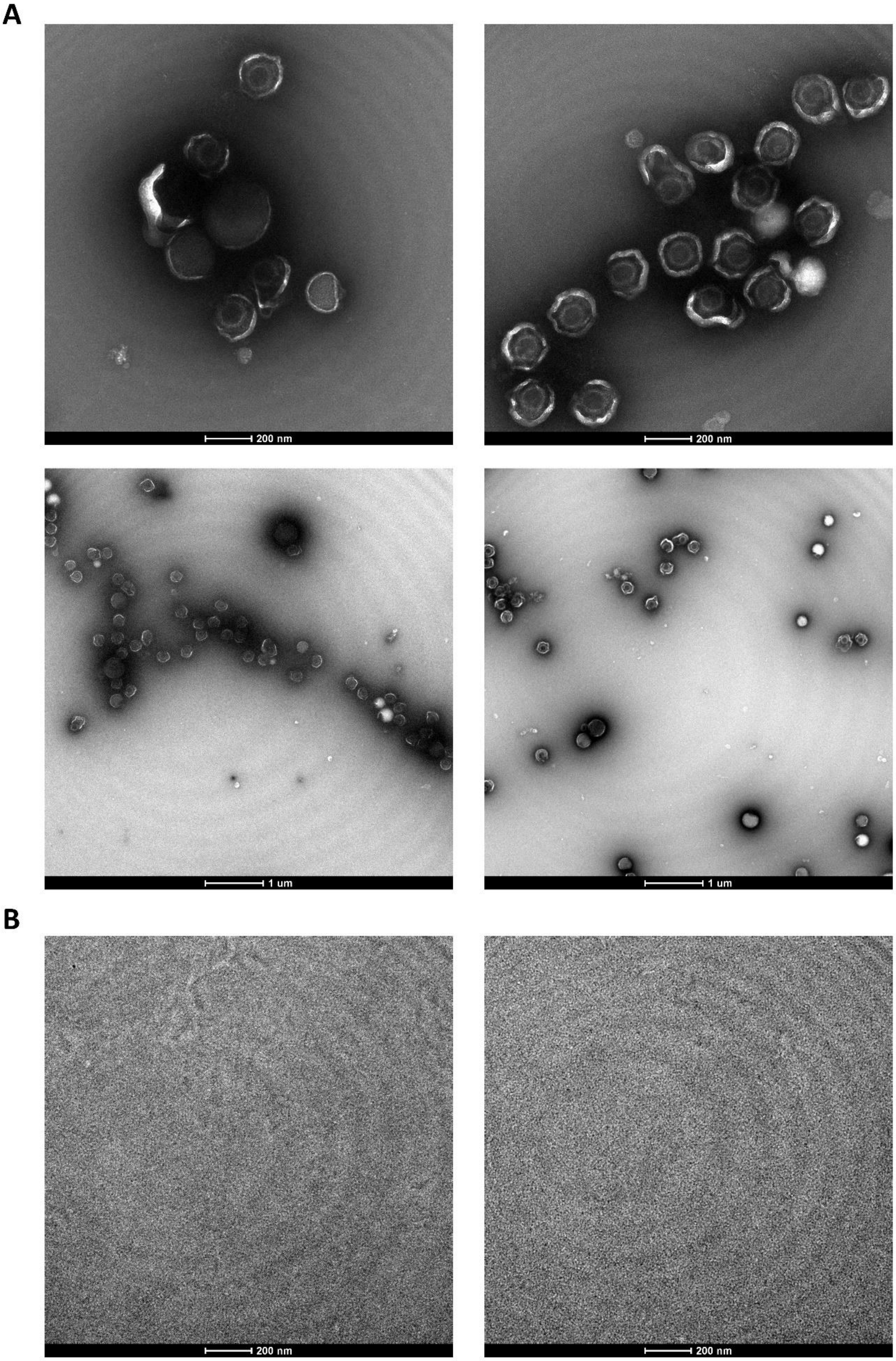

Figure S8. Transmission electron microscopy micrographs of the 25,000xG pellets from HCMV-infected HFF cells (A) and uninfected HFF cells (B).

Micrographs are representative of 2 independent experiments.

Supplementary Figure 9

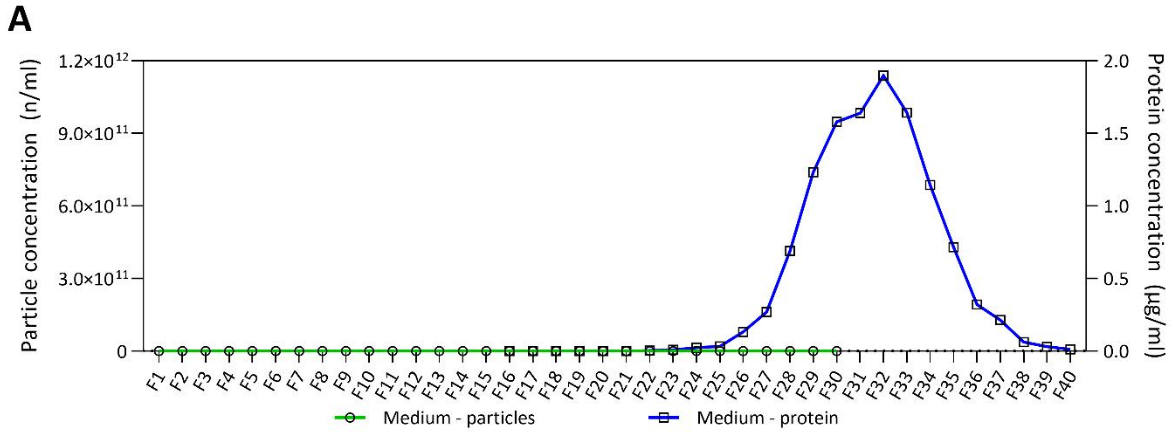

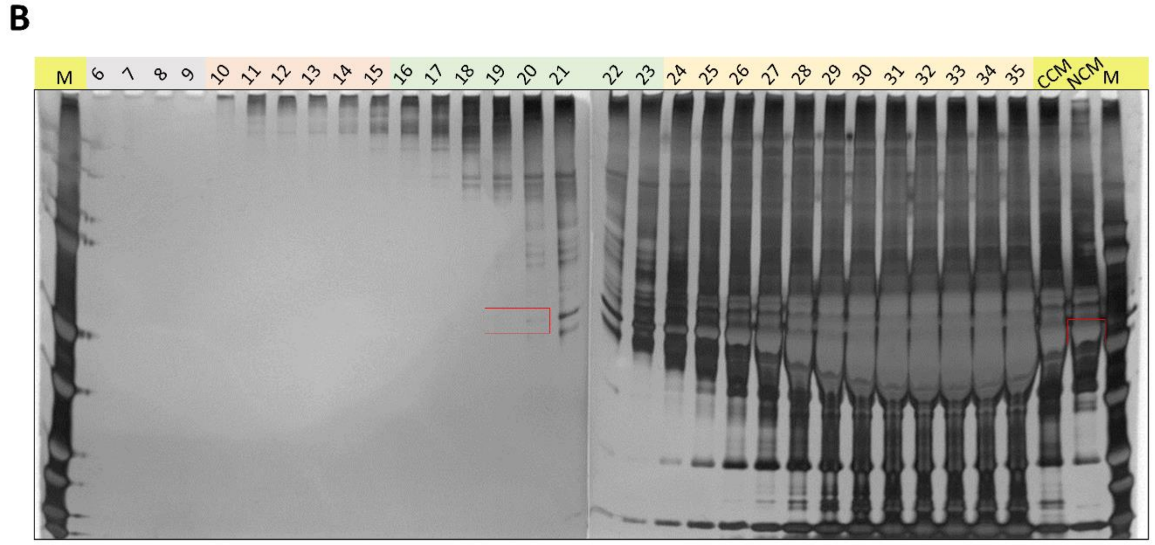

Figure S9. Analysis of non-conditioned medium SEC fractions by nanoparticle tracking analysis (NTA) and Bradford protein assay (A), and uninfected HFF SEC fractions by silver staining (B).

Supplementary Figure 10

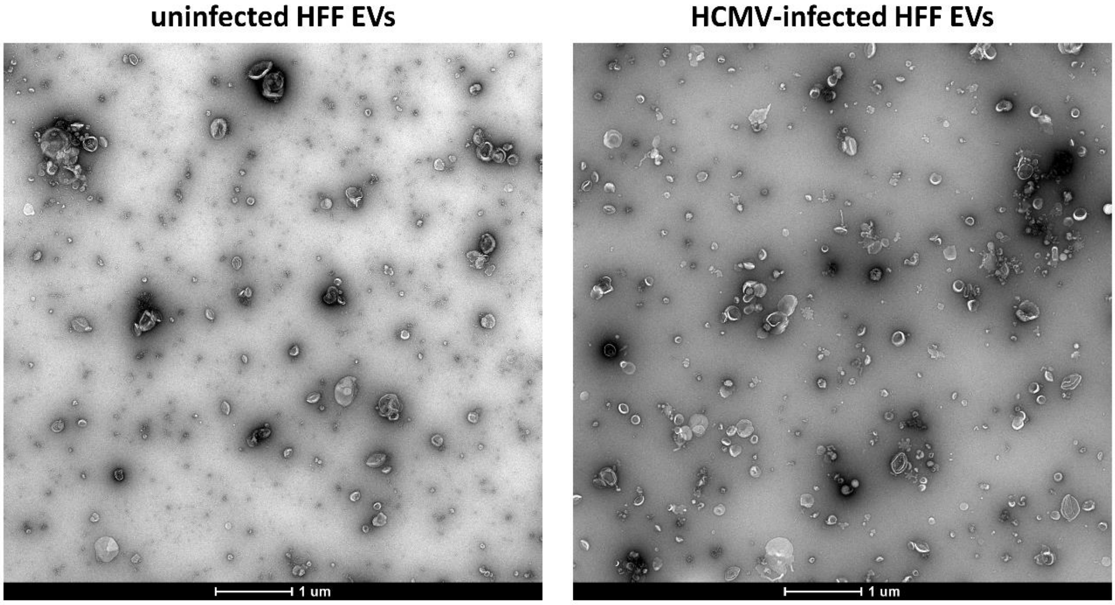

Figure S10. Widefield TEM micrographs of purified uninfected and HCMV-infected HFF EVs.

Supplementary Figure 11

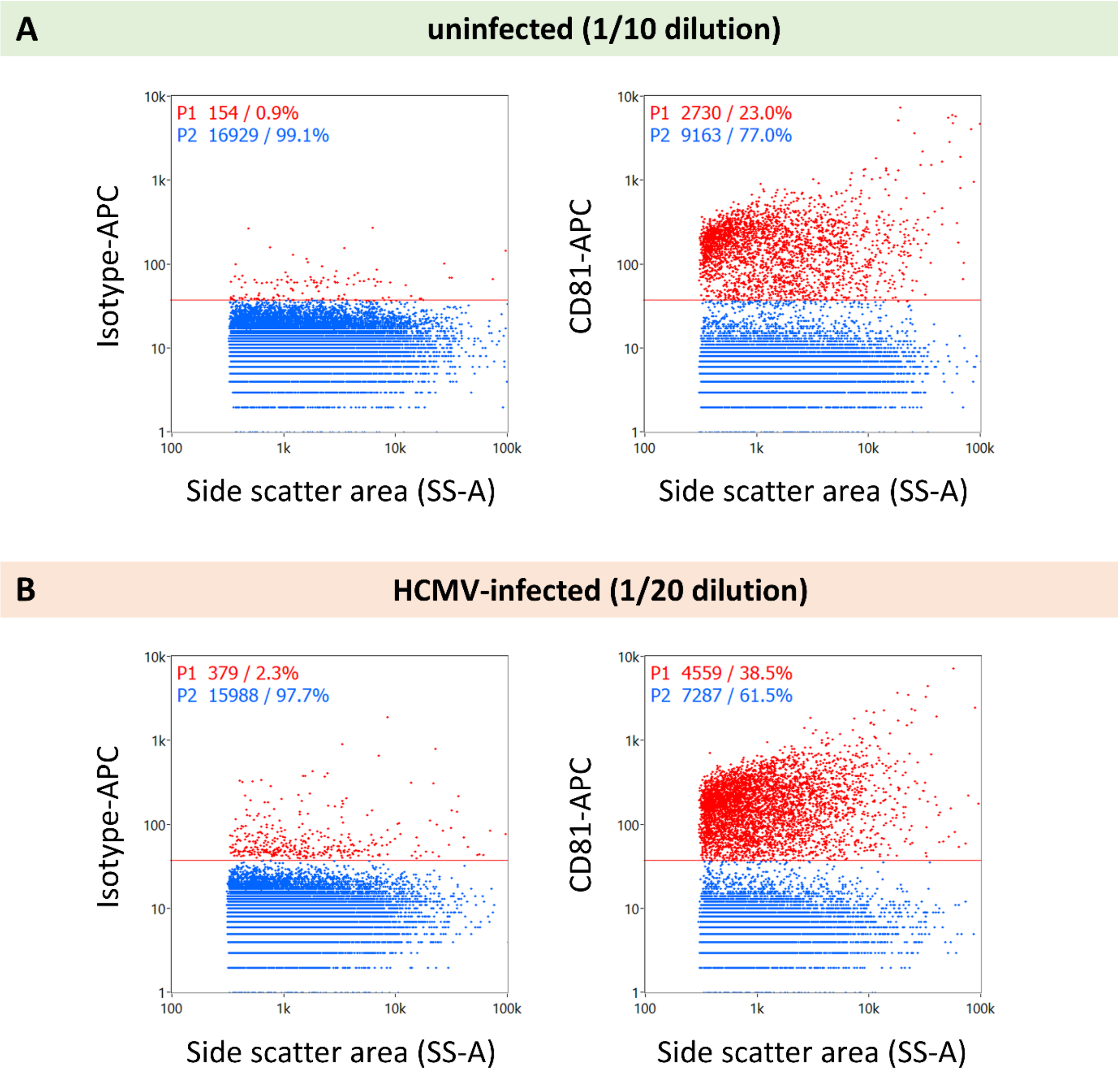

Figure S11. nFC analysis of CD81 on purified EVs from uninfected (A) and HCMV-infected (B) HFF cells.

EVs were labelled with a recombinant antibody against CD81, along with a matching isotype control, for 1 hour at room temperature. Excess antibody-fluorophore was removed by SEC and particles analyzed by nFC. Particles were excited at 635 nm and emitted fluorescence measured through a 670±15 nm bandpass filter.
