# Supplementary material for: Nano‐Flow Cytometry‐Guided Discrimination and Separation of Human Cytomegalovirus Virions and Extracellular Vesicles
Source: J Extracell Vesicles. 2025 May 2;14(5):e70060. doi: 10.1002/jev2.70060 (PMC12046292; doi:10.1002/jev2.70060)
Supplement: Supplementary file 2 — Supporting Information [file JEV2-14-e70060-s003.docx]

| **Requirement** | **Please Include Requested Information** |
| --- | --- |
| 1.1. Purpose | To discriminate human cytomegalovirus virions, dense bodies, and extracellular vesicles by nano-flow cytometry (nFC) following labelling of nucleic acid. |
| 1.2. Keywords | Cytomegalovirus; extracellular vesicles; nano-flow cytometry. |
| 1.3. Experiment variables | Extracellular particles obtained from uninfected or human cytomegalovirus-infected human foreskin fibroblasts (HFFs) were fixed with paraformaldehyde (4%) and labelled with nucleic acid dyes in DPBS buffer, before being analyzed on the flow nanoanalyzer (nanoFCM). Samples were analyzed at the sampling pressure of 1.0 kPa for 1 minute by excitation at 488 nm and 638 nm. Side scatter data was collected through a 488±5 nm filter, and fluorescence data through 525±20 nm and 670±15 nm bandpass filters. |
| 1.4. Organization name and address | Organization: Institute of Reproductive and Developmental Biology (IRDB), Department of Metabolism, Digestion and Reproduction, Faculty of Medicine, Imperial College London  Address: Du Cane Road, London W12 0NN, United Kingdom |
| 1.5. Primary contact name and email address | Vladimir Bokun - v.bokun20@imperial.ac.uk |
| 1.6. Date or time period of experiment | 2020-2024 |
| 1.7. Conclusions | Labelling of nucleic acid contents of extracellular particles released by HCMV-infected HFF cells enables discrimination and specific quantification of the HCMV virions, extracellular vesicles, and dense bodies. |
| 1.8. Quality control measures | The instrument is adjusted and calibrated with each use using quality control nanospheres (purchased from nanoFCM), and blank samples. Additionally, side scatter measurements are calibrated using silica nanospheres of known diameters. |
| 2.1.1.1. (2.1.2.1., 2.1.3.1.) Sample description | Samples include extracellular particles produced by uninfected HFF cells or those infected with human cytomegalovirus. These were in the form of clarified conditioned medium, size exclusion chromatography-purified extracellular particles, or samples enriched in virions or extracellular vesicles obtained by high-speed centrifugation. All samples were fixed with 4% paraformaldehyde and diluted at least 10-fold in DPBS prior to analysis. |
| 2.1.1.2. Biological sample source description | Human foreskin fibroblasts (HFFs) were obtained from ATCC and grown in DMEM supplemented with exosome-depleted fetal bovine serum (10%) and antibiotics. Cells were left uninfected or were infected with the human cytomegalovirus (HCMV) before conditioned medium containing extracellular particles including viral particles and extracellular vesicles was collected and clarified by centrifugation, and then optionally subjected to size exclusion chromatography or high-speed centrifugation.  The human cytomegalovirus (HCMV) strain Merlin R1111 was obtained from Dr Richard Stanton’s laboratory at University of Cardiff School of Medicine. This HCMV strain was derived from clinical material and captured in a bacterial artificial chromosome (BAC) before being transfected into permissive cells for reconstitution of mature infectious virus stocks. |
| 2.1.1.3. Biological sample source organism description | NA |
| 2.1.2.2. Environmental sample location | NA |
| 2.3. Sample treatment description | Samples to be analyzed by flow nFC were fixed with 4% paraformaldehyde for at least 5 minutes and then diluted in DPBS containing nucleic acid dyes SYTO 13, SYBR Green I or SYBR Safe. |
| 2.4. Fluorescence reagent(s) description | SYTO 13, SYBR Green I and SYBR Safe are nucleic acid-interacting fluorogenic dyes which are used for visualizing DNA and RNA in various biological samples. SYTO 13 is characterized by equal quantum yields when bound to DNA and RNA, while SYBR Green I is reported to preferentially interact with DNA. Lastly, SYBR Safe is used for staining DNA and RNA. |
| 3.1. Instrument manufacturer | nanoFCM |
| 3.2. Instrument model | Flow Nanoanalyzer |
| 3.3. Instrument configuration and settings | The instrument is equipped with 488-nm and 638-nm lasers and 488/10, 525/40, 580/40, 670/30 and 710/40 filters. Side scatter and fluorescence signals are detected by 3 single photon counting modules (SPCMs). In our experiments, we measured side scatter by excitation at 488 nm at 10 kW through the 488/10 filter, and fluorescence by excitation at 488 nm and 638 nm (both at 10 kW) through 525/40 and 670/30 filters, depending on the nucleic acid dye or fluorophore used. Sampling pressure was kept constant and experimental samples were analyzed under the same conditions as quality control nanospheres that were used for concentration measurements and side scatter calibration. |
| 4.1. List-mode data files | Data files generated by the instrument are of the .nfa format. These files were converted to .fcs format in the software that operates the instrument, using the ‘small signal’ thresholding setting. We deposited data to the Zenodo online data repository. The URLs are listed below:  - <https://doi.org/10.5281/zenodo.14270416>  - <https://doi.org/10.5281/zenodo.14270559>  - <https://doi.org/10.5281/zenodo.14270583>  - <https://doi.org/10.5281/zenodo.14270626>  - <https://doi.org/10.5281/zenodo.14270659>  - <https://doi.org/10.5281/zenodo.14270701> |
| 4.2. Compensation description | No compensation was necessary in our experiments. |
| 4.3. Data transformation details | NA |
| 4.4.1. Gate description | Rectangular gates were drawn on the human cytomegalovirus virions, dense bodies or extracellular vesicles, and the concentrations of these particle types determined. |
| 4.4.2. Gate statistics | Particles included in gates were processed for particle concentration, mean and median diameter with standard deviation, as well as median fluorescence intensity. |
| 4.4.3. Gate boundaries | Gates are shown in the figures. |
